# Plant recognition by *Trichoderma Harzianum* elicits upregulation of a novel secondary metabolite cluster required for colonization

**DOI:** 10.1038/s41598-025-33935-2

**Published:** 2026-01-20

**Authors:** Miriam Schalamun, Guofen Li, Wolfgang Hinterdobler, Dominik K. Großkinsky, Stéphane Compant, Assia Dreux-Zigha, Jennifer Gerke, Russell Cox, Monika Schmoll

**Affiliations:** 1 Center for Health and Bioresources, AIT Austrian Institute of Technology GmbH, Konrad Lorenz Strasse 24, Tulln, 3430 Austria; 2 Greencell, St Beauzire, 63360 France; 3 Institute of Organic Chemistry, Leibniz Universität Hannover, Schneiderweg 38, Hannover, 30167 Germany; 4 Department of Microbiology and Ecosystem Science, Division of Terrestrial Ecosystem Research, University of Vienna, Djerassiplatz 1, Vienna, 1030 Austria; 5 Present Address: MyPilz GmbH, Wienerbergstrasse 55/13-15, Vienna, 1120 Austria

**Keywords:** *Trichoderma harzianum*, *Hypocrea lixii*, Biocontrol, Plant protection, Plant-fungus interaction, Secondary metabolism, MAMP (microbe-associated molecular pattern), Interkingdom communication, PCA cluster, Microbiology, Molecular biology, Plant sciences

## Abstract

*Trichoderma harzianum* is a filamentous ascomycete frequently applied as biocontrol agent in agriculture. While mycoparasitism and antagonism of *Trichoderma* spp. against fungal pathogens are well known, early fungal responses to the presence of a plant await broader investigation. Analyzing early stages of plant-fungus communication we show that *T. harzianum* B97 chemotropically responds to a plant extract and that both plant and fungus alter secondary metabolite secretion upon recognition. We developed a strategy for omics-analysis simulating conditions of early plant recognition eliciting a chemotropic response in the fungus and found 102 genes to be differentially regulated, including nitrate and nitrite reductases. Additionally, the previously uncharacterized *P*lant *C*ommunication *A*ssociated (PCA) gene cluster was strongly induced upon recognition of the plant, comprises a palindromic DNA motif and was essential for plant colonization. The PCA-cluster is only present in the Harzianum clade of *Trichoderma* and closely related to a homologous cluster in *Metarhizium* spp. Horizontal gene transfer (HGT) was detected for PCA-cluster genes by plants, while the cluster in *T. harzianum* is likely under balancing or positive selection. Hence, the PCA-cluster mediates early fungus-plant chemical communication and may be responsible for the high potential of *T. harzianum* and closely related species for biocontrol applications.

**Supplementary Information:**

The online version contains supplementary material available at 10.1038/s41598-025-33935-2.

## Introduction

Natural environments harbor a complex community of microorganisms, which fulfill crucial tasks in the carbon cycle and can interact with plants as symbionts^1,2^ or pathogens^3^. Climate change and global warming are bringing increased disease pressure, abiotic stresses and promote invasion of plant pathogens in new habitats^4,5^. Hence, better understanding for knowledge based application of biocontrol agents and biostimulants is required^6,7^. Fungi as important members of soil microbiomes evolved elaborated mechanisms for dealing with their biotic and abiotic environment in terms of sensing and signaling mechanisms as well as strategies for effective competition and antagonism^8,9^. Fungal secondary metabolites are thereby of crucial importance for interactions with other microbes, animals and also with plants^10^.

The abilities of some fungi to antagonize and kill their competitors is applied for protection of plants against pathogens and fungi of the genus *Trichoderma* are among the most broadly applied for this purpose^11^. Consequently, this genus also dominates research towards mycoparasitism, plant protection and biocontrol of plant pathogens^12,13^. Fungi of the genus *Trichoderma*^14,15^ are typical inhabitants of the rhizosphere and are found in soils worldwide^16^. Several *Trichoderma* species are known as efficient biocontrol organisms and act as important symbionts with plants^17,18^. They are studied in detail for their capabilities in producing antibiotics, parasitizing other fungi – predominantly plant pathogens – and to compete with deleterious plant pathogens^13,16^. Beneficial *Trichoderma* strains further induce root branching, can increase shoot biomass and trigger systemic resistance as well as plant nutrient uptake^19^.

Among the most important functions for successful fungal plant interaction – beneficial or pathogenic - is the ability to colonize plant roots^13,20^. *Trichoderma* spp. are able to efficiently colonize plant roots, although they mostly remain at the outer layers of the plant tissue^13,19^. Nevertheless, also truly endophytic strains of *Trichoderma*^21,22^ are known to associate with plants, including also some *T. harzianum* strains^21,23^. Prior to colonization, but also thereafter, specific chemical communication – sensing, interpreting the relevance of chemical compounds and initiating an appropriate response – is required. Fungi communicate with their environment using a broad array of signals^24–26^. Chemical communication between fungi and plants is essential for interaction and diverse secondary metabolites are known to play an important role in this interplay^26^. Intriguingly, stressed plants were found to secrete specific compounds attracting beneficial fungi^27^. Overall, a considerable number of effectors^12^ and secondary metabolites^19^ including volatile organic compounds^28^ are already known to contribute to successful biocontrol. In a plant, recognition of beneficial fungi like *Trichoderma* spp. leads to metabolic changes^29^ and the onset of systemic resistance^30^. Acquisition of nutrients is supported by plant-fungus interactions, also if the requirements for mycorrhiza are not fulfilled.

Despite considerable knowledge on relevant signaling compounds secreted during interaction, one major question of the last decades was how fungi sense the presence of a plant in their environment. A seminal study on plant recognition by the fungal pathogen *Fusarium oxysporum* provided groundbreaking insights in this respect^31,32^. It was shown that this fungus chemotropically responds to the presence of a plant and that this response is dependent on a pheromone receptor of the fungus, which was shown to sense a peroxidase of the plant^32,33^. This research and the developed method open up new possibilities to study plant-fungus as well as other intra- and interkingdom interactions and their determinants^34^. At the same time, the involvement of a peroxidase in the sensing process suggests that the relevant compounds might be enzymatically modified prior to recognition, a mechanism shown previously for microbial interactions as well^35^.

The question of how the interaction between a fungus and a plant works is most important if the plant requires protection from a pathogen. A beneficial, protective interaction of fungi with plants is called biocontrol and gains increasing attention as ecological alternative to chemical pesticides. Biocontrol of plant pathogens^36^ is a complex mechanism: it involves processes from secretion of enzymes to production of secondary metabolites to mycoparasitism on the fungal pathogen^37^. *Trichoderma* spp. as well as other fungi applied in agriculture as plant beneficial agents produce a broad array of secondary metabolites^38,39^. Nevertheless, these fungi (except for a few more problematic species like *T. brevicompactum*) have a long history of safe application worldwide. No contamination of treated crops has been observed and hardly any negative effects on plants are known. Thereby, not only the fungi themselves, but also their secondary metabolites can be applied in agriculture^40^. Moreover, investigation of secondary metabolites of *Trichoderma* and their functions bears the opportunity to identify novel, bioactive compounds potentially useful in medicine and industry^41^. Considering the genomic equipment of *Trichoderma* for the task of biocontrol, evolutionary analysis revealed that the core genome of *Trichoderma* species comprises about 7000 genes and that a considerable number of genes crucial to their well-known functions in litter degradation or secondary metabolism, which are both relevant for biocontrol, was acquired by horizontal (or lateral) gene transfer (HGT)^14,42,43^. This successful sourcing of advantageous genes by *Trichoderma* is assumed to be corroborated by their capability of mycoparasitism, which brings them in contact with foreign DNA^42^. Such feeding activities along with physical association over prolonged periods of time, like for example an association of a fungus with a plant are known to facilitate HGT between eukaryotes^44^. Additionally, asexual development of fungi, involving unicellular spores, is in agreement with the “weak link hypothesis” for entry of foreign genes into eukaryotic genomes^45^. Acquisition of secondary metabolite clusters can be particularly beneficial for a biocontrol agent, due to their potential function in communication with the plant and/or fending of competitors or antagonizing pathogens. Nevertheless, the relevance of HGT in eukaryotes remains controversial^46,47^, and careful analysis is required to exclude for example sequencing artifacts jeopardizing the results^48^.

For this study on plant-fungus communication, *Trichoderma harzianum* B97^49^ was selected for its high efficiency in stimulation of plant growth, which indicates successful and beneficial interaction with plants. Moreover, the strain shows solubilization of phosphate and can alleviate abiotic stresses. Due to its proven efficiency in agricultural applications as biocontrol agent, it was the ideal isolate for studying its communication with a plant in more detail. We show that *T. harzianum* B97 chemotropically reacts to the presence of a plant and chemically communicates with a living plant as well. Moreover, we found that the ability of B97 to efficiently colonize plant roots depends on a secondary metabolite cluster specifically induced during early plant recognition, which is likely under balancing or positive selection.

## Results

### *T. harzianum *is an efficient colonizer of plant roots

**Fig. 1:**
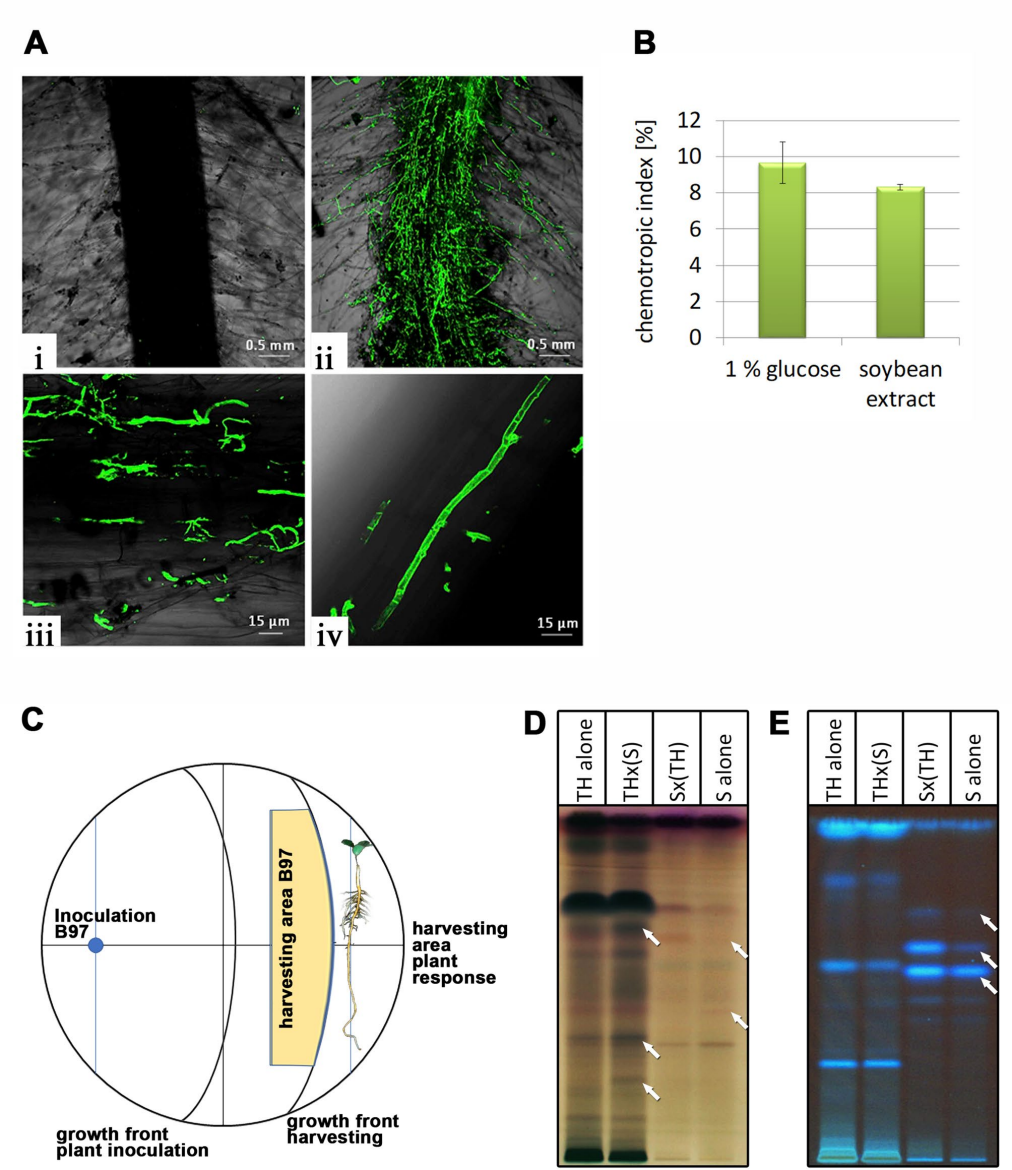
Assessment of *T. harzianum* B97 – plant interaction. (**A**) CSLM Microphotograph of uninoculated control roots of wheat (i) and wheat roots with *T. harzianum* B97 (ii-iv) at the root hair zone and stained with WGA-Alexa Fluor488^®^ showing B97 as green fluorescent colonizing wheat root hairs (iii) or the root surface (iv). (**B**) Chemotropic indices of *T. harzianum* B97 to the presence of 1% (w/v) glucose or root exudates of a soy plant. Analyses were done in biological duplicates, at least 400 germlings were counted per experiment. Chemotropic indices showed a statistically significant difference to background levels (p-value < 0.01). Error bars represent standard deviation. (**C**) Schematic representation of the experimental setup for analysis of plant-fungus communication. Plants were allowed to interact with the fungus for 13 h and harvesting was done before contact. Mycelium for investigation of the transcriptome was isolated from the mycelial growth front (“harvesting area B97”, yellow). For secondary metabolite analysis (SM) by HPTLC the agar slice including cellophane overlay from exactly the same area was excised. For analysis of the response of the plant an agar slice on the other side of the root (green area) was excised in order to avoid interference with fungal metabolites. For control plates the setup and positioning of harvesting areas was exactly the same. (**D**, **E**) HPTLC analysis of *T. harzianum* B97 alone on the plate (TH alone), *T. harzianum* B97 in the presence of the root of soy plant (THx(S)), the root of soy plant in the presence of the fungus (Sx(TH)) and the root of soy plant alone (S alone). Two different visualizations are provided (**D**: Visible light after anisaldehyde derivatization and **E**: Emission at 366 nm) and show differentially secreted metabolites between interaction partners alone and in combination. A representative analysis result is shown. For results of all three biological replicates, see supplementary file 2, Fig. S1.

*T. harzianum* B97 was isolated from agricultural soil in France^49^ and selected for its plant beneficial characteristics. Good colonization of plants by the fungus would indicate efficient communication with the plant^50^. Therefore, we studied, whether *T. harzianum* B97 would be able to efficiently colonize plant roots. We analyzed colonization after co-inoculation of wheat seedlings with *T. harzianum* B97. Staining of roots with wheat germ agglutinin (WGA)-AlexaFluor488 and confocal microscopy showed efficient colonization of roots by *T. harzianum* B97 (Fig. 1A).

### *T. harzianum *B97 shows chemotropic response to soybean root exudates

Recognition of the presence of the plant in the vicinity is crucial for initiation of interaction. Moreover, successful interaction with different plant species is a desirable trait for biocontrol agents. Hence, we tested interactions of the fungus with soybean roots in the following, which would show versatility of the strain in plant communication and complement the initial results gained with wheat (Fig. 1A). Recently, attraction of a *T. harzianum* strain to plant roots was shown^27^. Hence we wanted to test first, whether *T. harzianum* B97 chemotropically reacts to the presence of roots of soy plants using their root exudates as chemotropic agent. Optimization of the assay to *T. harzianum* B97 yielded an optimal working concentration of 0.0025% peptone from casein to support germination without inducing multipolarity. We tested the response of *T. harzianum* B97 to 1% (w/v) glucose, which resulted in a chemotropic index of 9.66 ± 1.15% and was hence in the range seen previously for fungi^32^. Subsequent analysis of the chemotropic response to soybean root extracts showed a chemotropic index of 8.32% ± 0.15%, representing low but clearly present response (Fig. 1B). This results further shows that the previously detected chemotropic response is not limited to the *Fusarium*-tomato interaction system, but a rather general response.

### Secretion of secondary metabolites changes in the presence of soy bean roots

As *T. harzianum* B97 clearly reacts to the presence of the plant, we wanted to test whether chemical communication is initiated as a consequence of recognition. We used conditions as close as possible to those applied in the chemotropic assay, with a low level of carbon source (0.1% (w/v) glucose) and minimal medium nutrients to support growth and still allow plant recognition (Fig. 1C). After 34 h of growth of *T. harzianum* B97, roots of soybean plants were placed in 3 cm distance of the fungal growth front and incubated for 13 h in the darkness to enable communication. To ensure that communication occurs via the medium or via volatile organic compounds, but not due to direct contact, we only used plates where fungus and plants remained without physical contact at the time of harvesting (Fig. 1C). Thereafter, agar slices were excised from the area covered by the fungus for assessing changes in fungal metabolite profiles as well as from the area opposite of the plant root to analyze alterations in metabolites secreted by the plant (Fig. 1C). Secondary metabolite patterns from the fungus grown without a plant and of the plant in the absence of the fungus under otherwise similar conditions were used as controls.

Indeed, after 13 h of exposure of the fungus to the plant, we observed additional bands appearing in the high performance thin layer chromatography (HPTLC) analysis, reflecting a reaction of the fungus to the plant (Fig. 1D,E). Also the plant secreted additional compounds upon detection of the fungus, which were not present in the assay without the fungus (Fig. 1D,E). Consequently, *T. harzianum* B97 initiates chemical communication with the soy plant roots within 13 h of co-cultivation.

### Transcriptome analysis of early stages of plant recognition

Having confirmed that plant recognition by *T. harzianum* B97 indeed occurs and elicits a two-way communication, we adapted transcriptome analysis to these conditions by covering the agar surface with cellophane to enable harvesting of the mycelium. Using the same setup as depicted in Fig. 1C enabled us to compare the situation of growth of the fungus alone on the plate versus changes in transcript levels due to addition of a soy plant on the plate. Importantly, cellophane was used in all conditions analyzed for the transcriptome experiment in order to avoid artifacts due to consumption of cellophane (for example mis-regulation of cellulase genes) as a carbon source by the fungus. Due to the high similarity of *T. harzianum* B97 with the previously sequenced reference strain *T. harzianum* CBS226.95^42^ we refer to protein IDs from the respective public genome database at JGI (https://mycocosm.jgi.doe.gov/Triha1/Triha1.home.html) in the following.

**Fig. 2:**
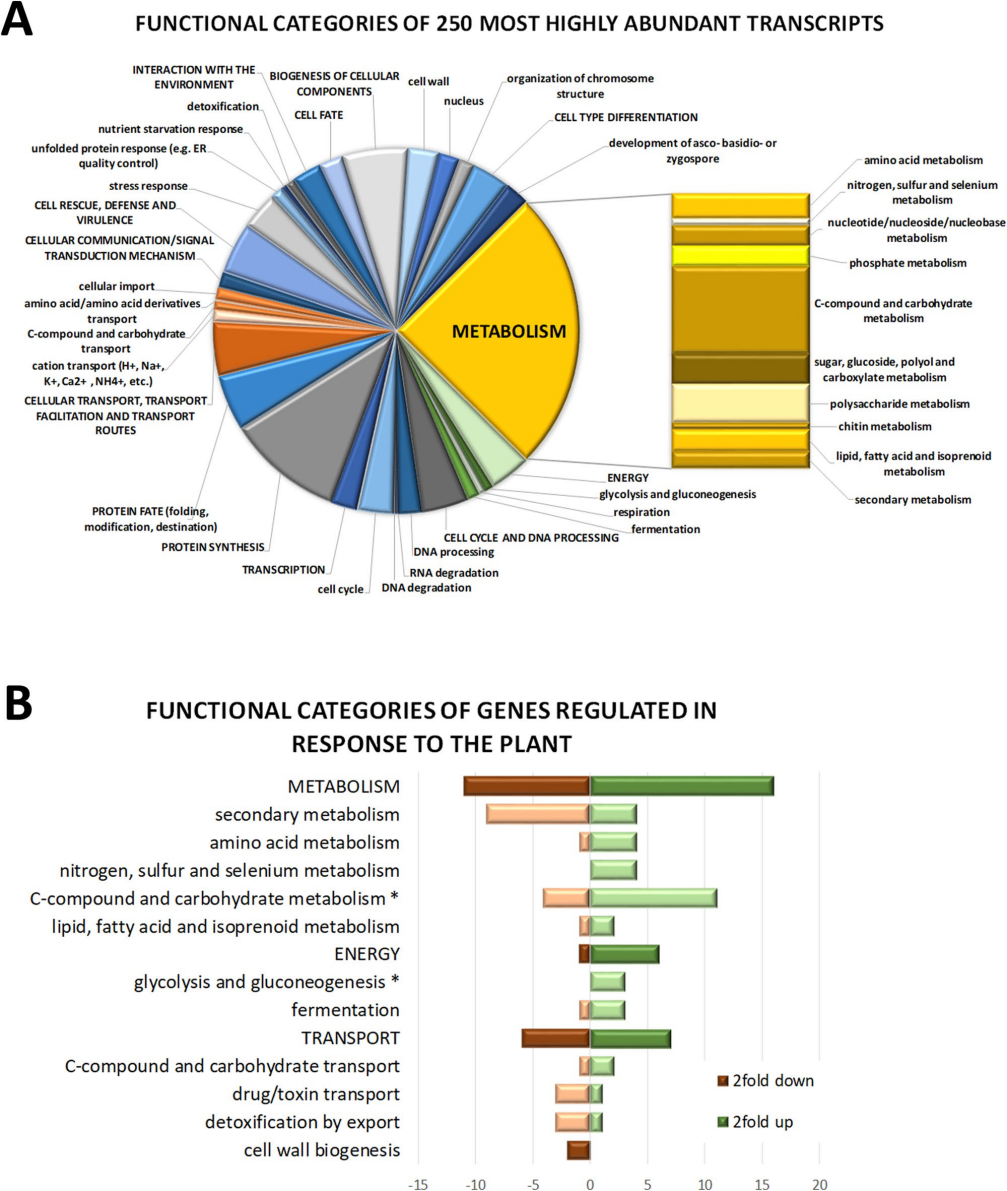
Functional analysis of gene expression in *T. harzianum* B97. (**A**) Functional categories represented among the 250 most abundant transcripts under the conditions of simulated chemotropic response. (**B**) Major functional categories assigned to genes differentially regulated in the presence of a soy plant. Significantly enriched categories are marked with an asterisk.

Since transcript levels reflect investment of resources for functions important under a certain condition, we checked which functions were represented under the conditions used for secondary metabolite screening and transcriptome analyses. Functional category analysis of the 250 genes with highest transcript levels under the applied conditions showed considerable investments in metabolic functions, energy production and transport among others (Fig. 2A).

Besides the expected enrichment in metabolic functions, energy metabolism and carbohydrate metabolism, we also found that the highest levels of transcript abundance were enriched in functions in stress response (p-value 1.96e-03) and unfolded protein response (p-value 7.89e-04). Additionally, also polysaccharide metabolism was significantly enriched in this highly transcribed gene set (p-value 2.49e-11), with the homologues of the cellobiohydrolases *cbh1* and *cbh2* comprised in this group (which may in part be due to the presence of cellophane in this assay). These patterns reflect that the chosen conditions indeed represent an environment of low nutrient availability inducing cellobiohydrolases likely due to starvation. Alternatively, regulation of cellobiohydrolases may be elevated due to the availability of cellulosic material present in the cellophane. Nevertheless, these data clearly show that cellulases are not repressed by the low concentration of glucose in the medium, hence not perturbing low nutrient conditions. Consequently, this condition closely resembles the conditions present in the chemotropic assay.

### Specific gene regulation in the presence of a plant

Comparison of genes differentially regulated between growth alone on the plate and in the presence of a plant confirmed that our experimental setup captured a very early specific stage of plant recognition by *T. harzianum* B97 and likely represents the onset of communication. In total, only 102 genes were significantly (p-value < 0.01) regulated more than 2fold (41 down, 61 up) upon recognition of the plant (supplementary file 1), which share functions in energy production, metabolism and transport (Fig. 2B). Genes upregulated in the presence of the plant were enriched in functions of C-compound and carbohydrate metabolism (p-value 1.04e-03), glycolysis and gluconeogenesis (p-value 4.09e-03) and electrochemical potential driven transport (p-value 4.00e-03). Interestingly, the gene set down-regulated in the presence of the plant is enriched in functions in secondary metabolism (p-value 5.03e-04) and drug/toxin transport (p-value 4.26e-03).

Specifically, we detected a nitrate reductase (Triha_507858) and a nitrite reductase gene (Triha_507859) to be up-regulated 17fold or 4fold, respectively upon plant recognition. These genes represent the homologues of the *Aspergillus nidulans* genes *niiA* and *niaD*, which share a bidirectional promotor and play important roles in nitrogen uptake and metabolism^51,52^. However, the putative homologue of *crnA*, the major facilitator superfamily (MFS) transporter gene associated with this cluster in *A. nidulans* (Triha1_142220) and the major transcription factor genes responsible for regulation of nitrogen metabolism, *areA* (Triha1_451) and *areB* (Triha1_70872), are not significantly differentially regulated under these conditions.

Considering fungus-plant interaction, also the more than 5fold up-regulation of Triha_398864, encoding a homologue of Epl1/Sm1 is interesting. These ceratoplatanin-like proteins play a role in colonization of plant roots and as effectors^53^. In *T. harzianum*, Epl1 regulates virulence of the plant pathogen *Botrytis cinerea*, mycoparasitism as well as plant immunity at early stages of root colonization^54,55^, which is in perfect agreement with our hypotheses.

Furthermore, transcript abundance of two predicted protease genes (Triha1_541862; 5.3fold down and Triha1_98848; 2.2fold down) is decreased in the presence of plant roots. As two putative terpene synthase genes, *tps1* (Triha1_497584; 2.8fold up) and *tps11* (Triha1_523651; 3.1fold up) are up-regulated, a role of terpenoid compounds in plant interaction is worth further investigation. Additionally, an as yet uncharacterized non-ribosomal peptide synthase (NRPS, Triha_155805) and a putative polyketide synthase (PKS, Triha_546993) are more than 2 fold upregulated upon plant sensing.

A further, strongly upregulated gene (32fold) is Triha1_36398, which is still uncharacterized and its encoded protein comprises no known domains. However, analysis of putative protein interaction partners using the homologue of this protein in *T. reesei* using the STRING database^56^; https://string-db.org; version 11.5) suggests a connection to a predicted ferric reductase, which fits to its genomic vicinity next to a putative ferric reductase (Triha1_76871) in *T. harzianum*, which is also strongly upregulated and fits to the general picture of gene regulation in B97 upon plant recognition.

We conclude that the recognition of a plant in the environment causes *T. harzianum* to modulate secondary metabolism, but to also elevate certain metabolic capabilities, notably also nitrogen metabolism which might be beneficial for nutrient exchange with a plant. Although functions in C-compound and carbohydrate metabolism are enriched among up-regulated genes, this gene set does not include the common plant cell wall degrading enzymes. The high expression level of cellulases detected in all analyzed samples (see above) is not significantly altered upon recognition of a plant.

### A secondary metabolite cluster strongly up-regulated upon plant recognition

**Fig. 3:**
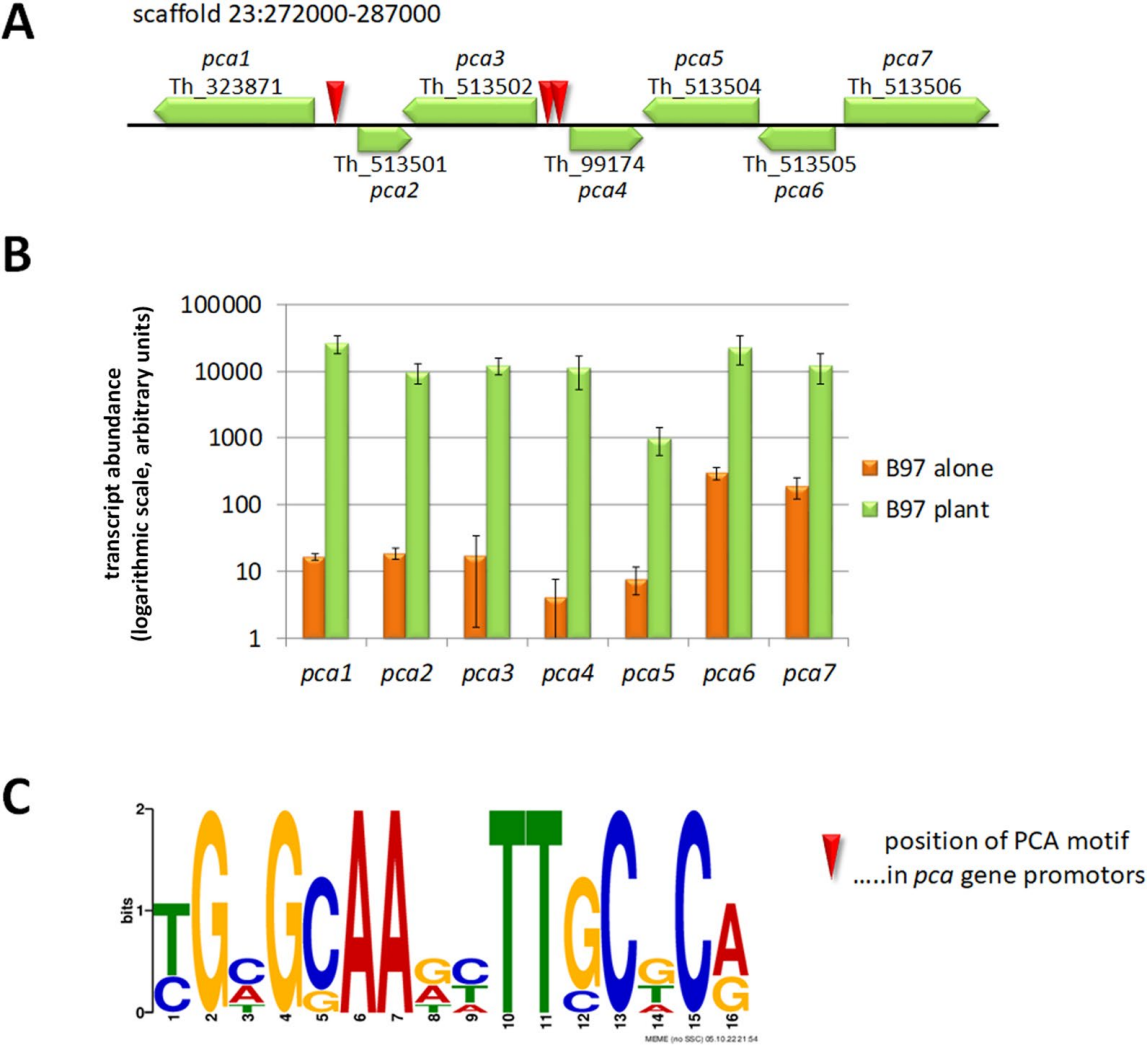
Schematic representation of the PCA cluster (**A**) and its regulation upon recognition of the plant (**B**). (**A**) Localization of *pca*-genes in the *T. harzianum* genome (JGI mycocosm; https://mycocosm.jgi.doe.gov/mycocosm/home). Approximate position of the PCA-DNA motif is shown with red triangles. (**B**) RPKM values of transcript levels of *pca* genes upon growth alone on the plate (orange bars), where transcripts were at very low basal levels (logarithmic scale is shown) compared to transcript levels in the presence of a plant with green bars representing transcript abundance upon recognition of the plant. Values represent means of three biological replicates and error bars show standard deviations. In all cases differential regulation is statistically significant (p-value < 0.01) (**C**) PCA motif as found in the potentially bi-directional promotors of *pca1*/*pca2* and *pca3*/*pca4*.

Despite the low number of regulated genes, we still found a strongly regulated gene cluster (Fig. 3A,B), which is silent when the fungus is growing alone, and strongly induced upon recognition of the plant with up to 1000-fold upregulation (Fig. 3B; supplementary file 1). This gene cluster has a size of 15 kb and is located on scaffold 23: 272 000–287 000 in the reference strain *T. harzianum* CBS226.95 and comprises all seven genes present in this area. We termed the cluster *P*lant *C*ommunication *A*ssociated (PCA) cluster which is comprised of Triha1_323871/*pca1*, Triha1_513502/*pca2*, Triha1_513502/*pca3*, Triha1_99174/*pca4*, Triha1_513504/*pca5*, Triha1_513505/*pca6* and Triha1_513506/*pca7*. None of the genes in the cluster was previously characterized.

Interestingly, also a further putative ferric reductase gene related to *pca1* is strongly up-regulated upon plant recognition (Triha1_76871; 21.2fold) as is a copper transporter gene closely related to *pca2* (Triha1_83588; 30.8fold). Both genes are not located in the genomic vicinity of the PCA cluster.

A SNP analysis of differences between *T. harzianum* B97^49^ and the publicly available sequence of *T. harzianum* (*sensu stricto*) CBS226.95^42^ revealed no intragenic, no intergenic and no nonsynonymous SNPs in the region of the PCA cluster. Only one synonymous SNP was detected in 513506 and one in 513501 in the 5’ UTR, hence strengthening the identification of *T. harzianum* B97 as *sensu stricto*. Consequently, we will refer to the *T. harzianum* CBS226.95 protein IDs and sequences hereafter.

In order to gain information on the potential function of the PCA cluster, we performed domain analysis and checked homologous genes in other fungi. PCA1 comprises a NADPH oxidase domain (cd06186) catalyzing the generation of reactive oxygen species (ROS) as well as a ferric reductase like transmembrane component (pfam01794) and is related to *A. fumigatus* FRE7, which is regulated by VeA^57^, upon response to Fe starvation^58^ and during hypoxia^59^. PCA2 contains a copper transporter domain (pfam04145), which may be involved in oxidative stress protection or pigmentation and is related to putative low affinity copper transporters in *Aspergilli*. PCA3 is a member of the transferase superfamily (cl23789), which comprises enzymes that catalyze the first committed reaction of phytoalexin biosynthesis, but also trichothecene 3-0-acetyltransferase. PCA3 represents a homologue of the *Fusarium* trichothecene 3-O-acetyltransferase Tri101. This enzyme is known to have a function in self-protection of trichothecene producing fungi like *Fusarium pseudograminearum*^60^. Although trichothecene production was reported for *Trichoderma*^61,62^, predominantly in the Brevicompactum clade^63^ comprising *T. brevicompactum* or *T. arundinaceum*, but not for species of other clades, we checked for the presence of genes associated with trichothecene production. Besides PCA3/Tri101, the *T. harzianum* genome comprises homologues of the trichodiene oxygenase TRI4 (Triha1_99853), the transcription factor TRI6 (Triha1_514309, distant relationship), the transcription factor TRI15 (Triha1_1233) and the isotrichodermin C-15 hydroxylase TRI11 (Triha1_535230). None of these genes is regulated upon recognition of a soy plant in our experiment (supplementary file 1).

PCA4 contains an NAD/NADP octopine/nopaline dehydrogenase (pfam02317) domain as well as a glutamate synthase or related oxidoreductase domain (cl28234), which may be involved in amino acid transport and metabolism. PCA4 has no homologues in *Aspergilli*. PCA5 is a major facilitator superfamily transporter (pfam07690), related to a cycloheximide resistance protein. PCA6 belongs to the superfamily of S-adenosylmethionine-dependent methyltransferases, class I (cl17173). PCA7 has a Cytochrome P450 domain (cl12078), which may be involved in the degradation of environmental toxins. It is relatead to the *Fusarium* isotrichodermin C-15 hydroxylase Tri11, but is no direct homologue according to reciprocal best hit analysis. In contrast to *Trichoderma brevicompactum*^62^, *T. harzianum* does not comprise a trichodermin cluster in its genome, as also outlined above. The homologue of PCA7 in *A. nidulans*, STCF, is a putative sterigmatocystin biosynthesis P450 monooxygenase with a predicted role in sterigmatocystin/aflatoxin biosynthesis.

Due to the tremendous up-regulation of the PCA cluster upon plant recognition, we were interested whether the genes of this cluster or their homologues in *T. virens* (which has the complete PCA cluster) are regulated in the presence of a plant as well. Therefore, we evaluated and/or re-analyzed available transcriptome data from earlier studies with maize or tomato, which however were carried out with different growth conditions^64–68^. Moreover, it cannot be excluded that regulation of the PCA genes is host plant specific and differs between maize or tomato and the soybeans used in our study. In most cases, the *pca*-genes and their homologues in *T. virens* were not significantly regulated (> 2fold, p-value < 0.05) during plant colonization and were not detected in the secretome associated with plant contact. Only in one study^65^, down-regulation of four of the seven PCA cluster genes was detected in a hydroponic interaction system in the colonization phase. (for details see supplementary data 1, supplementary file 2). Hence, we conclude that the PCA-cluster is particularly important during early plant recognition under conditions simulating solid phase interaction with the plant, but unlikely to be relevant in later phases of fungus-plant interaction.

### The PCA cluster region comprises multiple occurrences of a novel DNA motif

Due to the striking up-regulation of the PCA cluster genes upon plant recognition, we were interested whether a common DNA motif might be present in the promotors of the genes. To this end, we analyzed the promotors of all *pca* genes for promotor motifs using MEME. Interestingly, we found a palindromic motif 5’YGHGSAADHTTSCDCR 3’ (Fig. 3A, C) in the intergenic regions between *pca1* and *pca2* as well as *pca3* and *pca4*. This motif is present twice in each region and was not yet characterized in fungi. Scanning the JASPAR database (https://jaspar.elixir.no/analysis) for transcription factors potentially binding to this sequence unfortunately yielded no result. Screening of the whole genome of *T. harzianum* CBS226.95 yielded 13 additional appearances of this motif in the vicinity of genes with mostly unknown functions, albeit in some cases not up-stream of the ATG, but inside the open reading frame (supplementary file 1). However, none of these motifs resides upstream of a gene or within a gene cluster regulated by the presence of a plant as detected in this study. Hence the PCA motif supposably has a specific function during early plant recognition and/or for sole regulation of the PCA cluster.

### The PCA cluster is required for plant root colonization

**Fig. 4:**
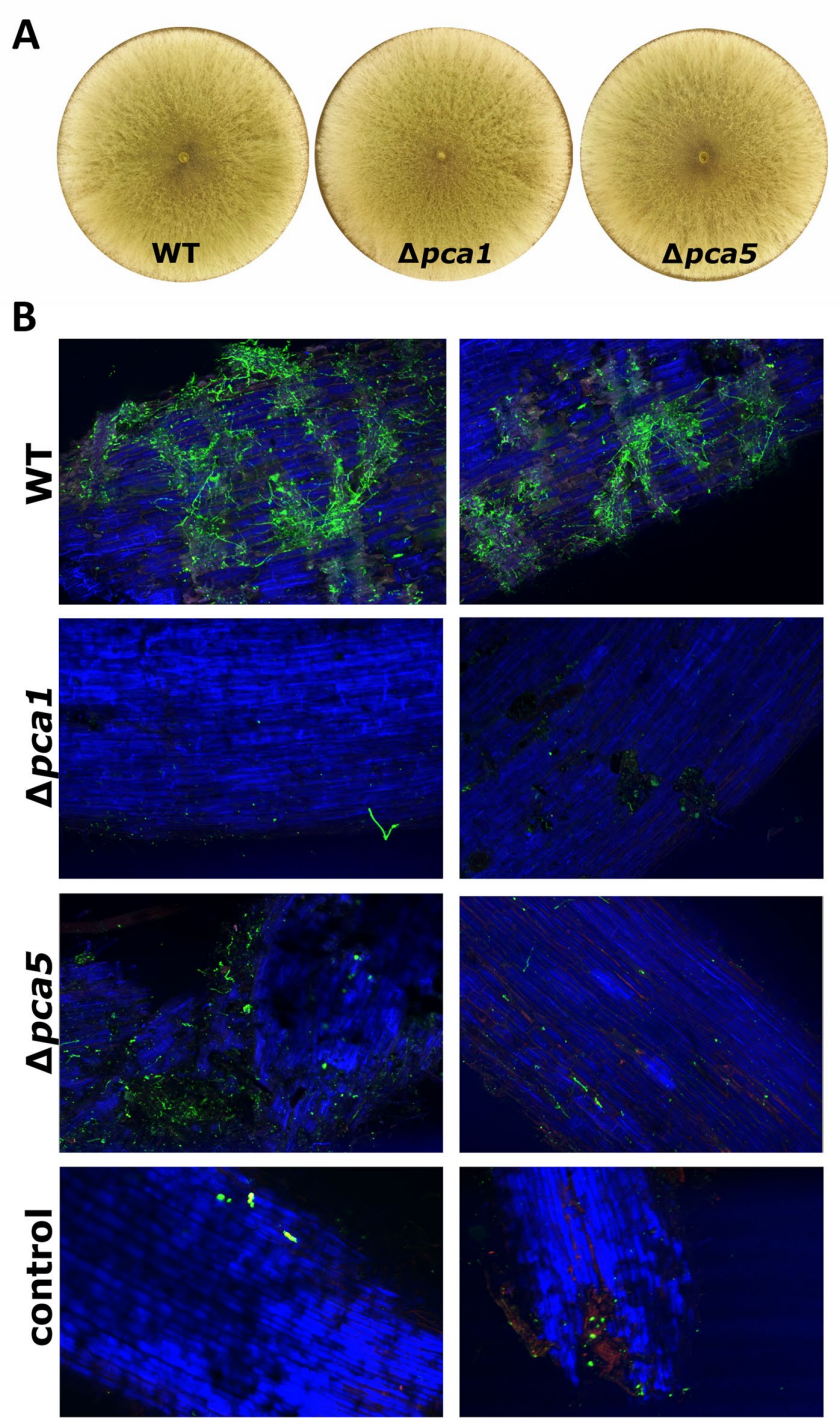
Colonization of soybean roots by *T. harzianum* B97 wildtype and mutant strains Δ*pca1* and Δ*pca5*. (**A**) Phenotype of recombinant strains (Δ*pca1* and Δ*pca5*, representative plates are shown) grown for 72 h in light on malt extract media. (**B**) Confocal microscopy images of soybean roots colonized with wildtype (WT), Δ*pca1* and Δ*pca5.* Images of two independent replicates showing different regions of the roots are presented (replicate **A**, replicate **B**). The two panels for each mutant (left panel, replicate A: strains pca1_13Aa and pca5_28Aa; right panel, replicate B: pca1_4Ba and pca5_27Ba) show representative pictures of independent transformants, selected from at least three biological replicates each and several sites of the respective replicate. Uninoculated roots were used as control (“no fungus”). Fungal mycelia on the soybean roots were stained with WGA-Alexa Fluor488^®^. CLSM micrographs are showing B97 hyphae as green fluorescent colonizing the roots of wildtype but hardly detectable with both mutant strains.

We further asked whether the early and strong transcriptional response of the PCA cluster genes upon plant recognition would be predictive of a role in colonization, which is crucial for plant-fungus interaction and plant protection^26^. For construction of deletion strains we chose *pca1*, the putative NADPH oxidase, since previous work revealed an involvement of ROS – directly or indirectly – in plant-fungus interaction^32,33^. Additionally, we prepared a null mutant of *pca5*, encoding a transporter potentially involved in signal compound emission. Both mutant strains were viable and showed no striking growth defects (Fig. 4A).

We tested the ability to colonize plant roots by inoculating soybean seeds with the mutant strains or the wildtype and evaluated the presence of fungal mycelia on young roots after 8 days (Fig. 4B). Confocal microscopy was performed from at least three replicate assays and multiple sites per root with two independent deletion strains per mutation. Roots grown from uninoculated seeds were used as controls. This analysis showed that while the wildtype *T. harzianum* B97 efficiently colonized the root surface of soy plants in addition to those of wheat as shown in Fig. 1A, suggesting this strain as a potential colonizer of a broader spectrum of plant species. Neither Δ*pca1* nor Δ*pca5* were able to colonize the root grown from seeds inoculated with the mutant strains and these samples rather resembled the uninoculated control (Fig. 4). We conclude that the requirement of these two genes of the PCA cluster is representative for the importance of this cluster and its upregulation upon plant recognition for efficient colonization of plants by *T. harzianum* B97.

### The PCA cluster is specific to trichoderma and subject to balancing or positive selection

Because of the obvious importance of the PCA cluster for plant recognition and colonization, we were interested in its conservation and evolution in fungi. Since the genomic region of the PCA cluster in B97 did not comprise a notable number of SNPs in comparison with *T. harzianum* CBS226.95, we will refer to the region in the latter strain in our further analyses and descriptions.

**Fig. 5:**
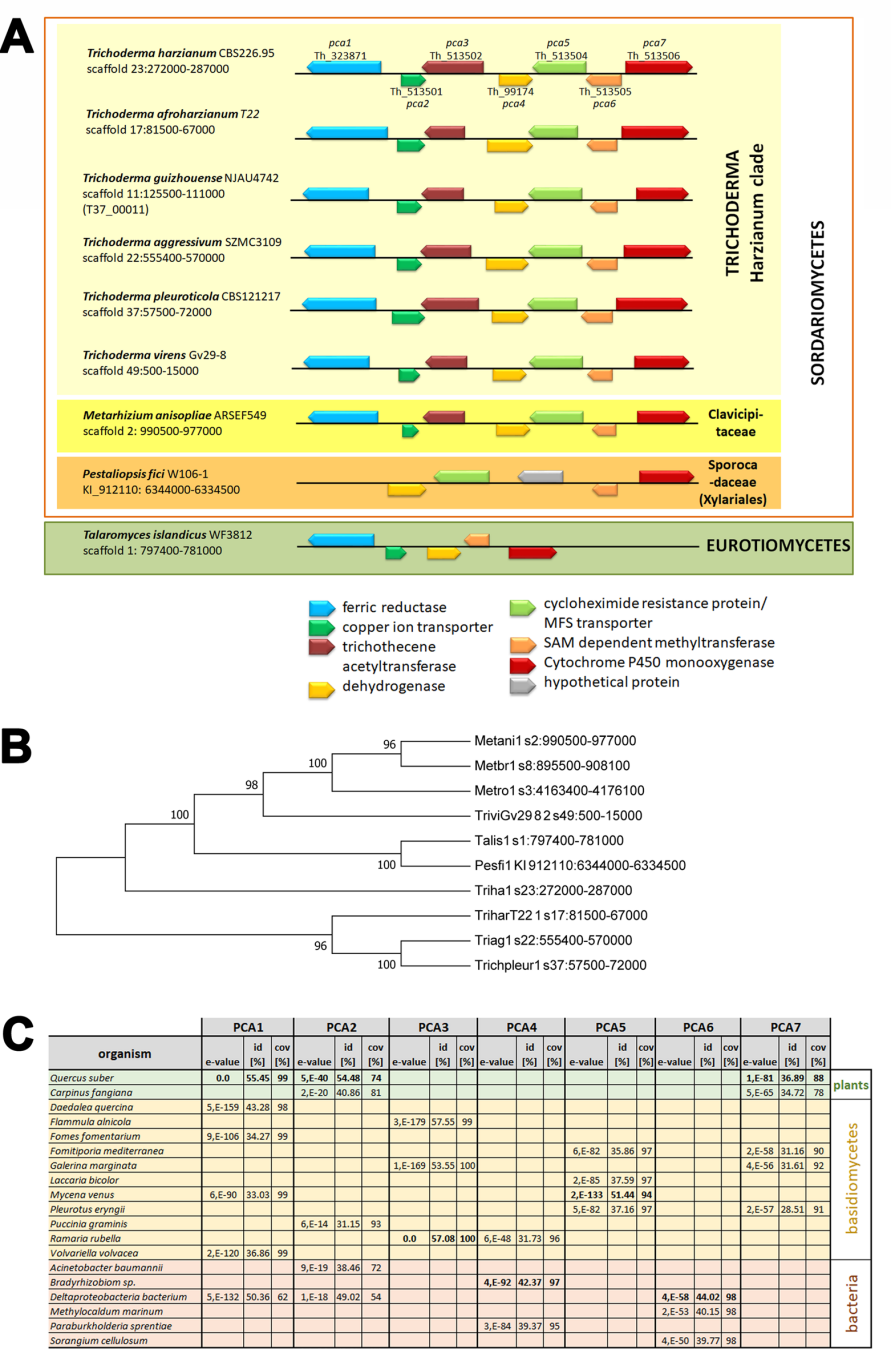
The PCA cluster, phylogenetic relationships and proteins related to its components. (**A**) Schematic representation of the clusters detected within selected representatives of *Trichoderma* spp. and outside the genus *Trichoderma*. For details on homology of the PCA-cluster homologues in these fungi, please see supplementary table S7 in supplementary file 2. (**B**) The evolutionary history was inferred by using the Maximum Likelihood method based on the Tamura-Nei model. The bootstrap consensus tree inferred from 1000 replicates is taken to represent the evolutionary history of the taxa analyzed. Branches corresponding to partitions reproduced in less than 50% bootstrap replicates are collapsed. The percentage of replicate trees in which the associated taxa clustered together in the bootstrap test (1000 replicates) are shown next to the branches. Initial tree(s) for the heuristic search were obtained automatically by applying Neighbor-Join and BioNJ algorithms to a matrix of pairwise distances estimated using the Maximum Composite Likelihood (MCL) approach, and then selecting the topology with superior log likelihood value. Sequences of *Metarhizium anisopliae* (Metani1), *Metarhizium brunneum* (Metbr1), *Metarhizium robertii* (Metro1), *Trichoderma virens* (TriviGV28_8_2), *Talaromyces islandicus* (Talis1), *Pestaliopsis fici* (Pestfi1), *Trichoderma harzianum* (Triha1), *Trichoderma afroharzianum* (TriharT22) and *Trichoderma aggressivum* (Triag1) originated from JGI mycocosm. (**C**) Blastp results of the respective protein sequences from *T. harzianum* B97 against the NCBI nr database with ascomycetes excluded. The top 100 hits for PCA1-7 were screened for most interesting similarities for this table.

After identifying the genomic area of the PCA cluster in *T. harzianum*, we performed a blastn analysis with all dikarya genome sequences available at JGI (2140 genomes). These genome sequences cover the group of Sordariomycetes (528 genomes) very well, including numerous strains of the genus *Trichoderma*, which would allow for association to the well-studied clades of the genus^69^. All other groups of fungi in JGI mycocosm outside dikarya^70^ were tested as well, but did not yield homologous sequences. Surprisingly, the search results did not reflect the expected relationships according to the known phylogeny of ascomycetes. Moreover, the cluster was not present in many *Trichoderma* species. Neither *Trichoderma* spp. outside of the Harzianum clade nor the common ancestor of the genus *Trichoderma*, *Escovopsis weberi*^71^ or closely related species such as *Fusarium* spp. had this cluster, as revealed by only partial coverage of the cluster sequence in the genomes. Rather it was scattered among some species in the Sordariomycetes. However, good coverage of the cluster area was detected for *Metarhizium* species as well as for *Pestaliopsis fici* (Xylariales^72^ and *Talaromyces islandicus* (Eurotiomycetes^73^ (Fig. 5A and supplementary data 2 in supplementary file 2). All genes present in these fungi had high homologies and very low E-values compared to the genes in T. harzianum CBS226.95 as revealed by blastp analysis (supplementary file 2, supplementary table S7). Using the respective nucleotide sequences covering the whole clusters for phylogenetic analysis and BLAST searches confirmed the close relationship of the cluster sequences (Fig. 5B, C). In case of *Metarhizium* spp. we found coverage of the cluster for *M. anisopliae*,* M. robertii* and *M. brunneum*, representing the generalist species of the genus^74^, but not in intermediate or specialist species, which is also the case for other secondary metabolite clusters in specialist species of *Metarhizium*^74^. Interestingly, the PCA cluster was not detected previously in *Metarhizium*^75^ and does not overlap with the well characterized secondary metabolite clusters responsible for production of destruxin, ferricrocin or other known toxins^74,75^. Furthermore, this finding suggested, that the PCA cluster was acquired by *T. harzianum* via horizontal gene transfer (HGT), likely from *Metarhizium* spp.

Detailed analysis of the ocurrence of the PCA cluster in *Trichoderma* spp. genomes available in JGI Mycocosm revealed that it is only present in those species of the Harzianum clade of *Trichoderma*, but not in the evolutionarily younger clades (supplementary data 2 in supplementary file 2). Consequently, we investigated whether the PCA cluster might have been acquired by *T. harzianum* by HGT, which was obviously not the case. However, we found that the similarity of plant gene products (but not bacterial gene products) encoded in plant genomes like *Quercus suber* or *Carpinus fangipana* is due to their acquisition of PCA1, PCA2 and PCA7 homologues from ascomycetes by HGT. Analysis of Tajima’s D as a measure of evolution indicated balancing or positive selection for the proteins within the PCA cluster. For details on these analyses see supplementary material (supplementary data 2 and Fig. S1 in supplementary file 2).

### Assessment of potential compounds associated with the PCA cluster

Phylogenetic analysis or blast searches did not reveal a cluster similar to the PCA cluster for which the associated compounds would have been determined. Antismash analysis of the genomic region spanning the PCA cluster did not yield a result, likely because the cluster does not comprise a “core” gene such as a terpene cyclase, PKS or NRPS. Since two genes in a genomic locus separate from the PCA cluster encode a PKS and an NRPS and both are somewhat up-regulated (see above), the cluster could be split with the core genes separated from the cluster. Antismash analysis of this separate locus indicated several possible products with 6-methylsalicylic acid reaching the highest similarity score.

To evaluate the hypothesis of a split cluster, we performed CBlaster analysis^76^ for identification of similar BGCs in other fungal genomes. In many cases, the species comprising the full or a partial cluster are pathogens like *Pyricularia* spp. or *Colletotrichum* spp. (supplementary file 3). While all 7 genes are present in part of *Trichoderma* spp. and in *Metarhizium*, many other species only comprise the genes encoding PCA4, PCA5 and PCA6, which hints at these proteins representing the core chemistry.

Comparison of the CBlaster BGC results using Clinker^77^ was performed to elucidate whether PCA-like clusters in other fungi potentially comprise core genes like such encoding PKS or NRPS as well. As this was not the case (supplementary file 4), it is unlikely that the PCA cluster is split and that the locus comprising an NRPS and PKS encoding gene are associated with the PCA cluster. However, this also leads us to the conclusion that the chemistry responsible for the compound formed by the proteins encoded in the PCA cluster is somewhat cryptic and the alternative that a plant metabolite might be modified by these proteins could be considered.

## Discussion

Recognition of plants by fungi is crucial if the actual cry for help in the form of root exudates^78^ is to be heard. Beneficial fungi of the genus *Trichoderma* positively impact plants at multiple levels, also using chemicals for achieving their effect^26^, but most importantly, they trigger the systemic immune response of plants^18,79^. Hence they are ideal organisms to answer such a cry for help. *T. harzianum* strains produce a number of secondary metabolites^80^ with interesting bioactivity including antifungal activity^81^, most of which are not yet assigned to specific biosynthetic gene clusters.

Although secondary metabolism and the gene clusters involved in biosynthesis of secondary metabolites are well studied in *Trichoderma* species^39,82^, the plant recognition specific PCA gene cluster we found in this study was not described before. Also the detected DNA motif, which is likely to be relevant for the regulation of at least part of the cluster genes is still unknown. The enormous extent of the induction of this cluster, which exceeds the changes in transcript abundance of all other regulated genes, indicates that communication with the plant is associated with this induction, which we confirmed with investigation of two crucial genes of the cluster (*pca1* and *pca5*). Moreover, the fact that the presence of a plant elicits a response in terms of secondary metabolite production is in agreement with the finding that the secondary metabolite pattern of *T. harzianum* B97 is altered in the presence of a plant. Notably, the mutual influence of fungus and plant happens prior to physical contact and hence we cannot exclude the involvement of volatile organic compounds (VOCs). However, we also found that the PCA cluster is predominantly relevant at the very early recognition stage, but presumably less so upon physical interaction at the contact or colonization stage.

The striking impact of deletion of members of the PCA cluster on colonization of soybean roots strongly supports a function of secondary metabolite(s) either associated with the cluster or modified by one of the encoded enzymes in plant-fungus communication. This early, pre-contact interaction likely initiates a positive reaction of the plant, which fosters colonization by the fungus, as the latter process does not happen anymore if parts of the cluster are removed. Such communication is vital for beneficial interkingdom-interactions, which was also shown for *Trichoderma*^26^. The beneficial effects of *T. harzianum* B97 on plant health are extensive, facilitating commercial application, and may involve an influence of secondary metabolites – including those of the PCA cluster – on innate immunity as shown previously for *Trichoderma*^30^. However, as root colonization is a prerequisite for efficient interaction, it can be concluded that the early stage recognition and communication represents the major function of the PCA cluster, rather than interaction upon contact or at stages thereafter. This hypothesis is further strengthened by the very early induction of the cluster, within only 13 h of proximity and without direct contact between plant and fungus. Although the interaction between plant and fungus could be direct by secreted compounds which are sensed and then initiate a response, also the possibility of modification of secreted compounds by the interaction partner (here plant or fungus) and sensing of a thereby specifically modified signal should be considered. Such modifications are common in organismic interactions for example between bacteria and fungi and here a “gain of function” modification would likely be applied^35^.

Considering the potential reason for the relevance of the transporter PCA5, our analysis of gene regulation patterns specific for early plant recognition by *T. harzianum* B97 had revealed a significant enrichment of genes involved in detoxification by export, which is likely achieved by transporters. Therefore, we investigated the role of PCA5 in plant interaction and indeed found that this transporter is crucial for colonization. This finding is in agreement with the hypothesis, that the chemical communication with the plant as initiated by induction of the PCA cluster is dependent on export of secondary metabolites serving as signaling molecules by PCA5. Alternatively, PCA5 may be involved in import of plant signaling compounds or even a modified fungal compound, which initiate beneficial communication by the fungus.

As for the contributions of the remaining cluster constituents, the PCA cluster comprises three genes putatively involved in biosynthesis or modification of secondary metabolites. Among the genes regulated in response to the presence of the plant, we found several more putative permeases and transporters, but no gene which might encode a transcription factor. Hence, it remains to be demonstrated, how the coordinated induction of the PCA cluster genes is achieved.

Considering the NADPH oxidase domain of PCA1 which is also essential for colonization, a relevance of an NADPH oxidase involved in ROS production for plant-fungus interaction was previously shown for *T. atroviride*^83^. For *F. oxysporum*, NADPH oxidase was found to be essential for chemotropic response to the presence of a plant^33^.

While we could clearly show that the plant and the fungus do interact, the compound formed by the PCA cluster proteins or with their contribution remains to be determined. Nevertheless, the predicted function of part of the proteins allows for an estimation of potential outcomes, which may support further analyses: PCA4, as an opine dehydrogenase/synthase, may be involved in condensing an amino acid (often arginine) with an alpha-keta acid such as pyruvate and reduction of the formed imine to a secondary amine. Such compounds are involved in Crown-Gall formation after *Agrobacterium tumefaciens* infection in plants and to some extent opine-related molecules serve as signaling compounds^84^. Moreover, such compounds could potentially be methylated and oxygenated by PCA6 and PCA3. Interestingly, fungi can use opines also as nitrogen sources^85^ and hence the up-regulation of *pca4* as well as the nitrogen metabolic genes homologous to *niiA* and *niaD* could constitute a coordinated response to nitrogen requiring conditions.

Another possibility is that the fungus may use the compounds to obtain metals from the host – which is corroborated by the presence of the ferric/cupric reductase PCA1 and the copper transporter PCA2 in the cluster. The recently reported mechanism involving opine-type proteins was so far only described for bacteria^86^. In the bacterial pathogen *Staphyllococcus aureus* the compound staphylopine (related to opines and derived from pyruvate) is involved in metal acquisition^87^ in a mechanism which includes specific recognition of the metal bound protein^88^. According to this hypothesis, an octopine-like compound would be made by PCA4, then methylated and acetylated and exported/imported to the host. Once the metal-bound molecule is re-imported, then the metal reductase would release the metal from the siderophore. While such a process would reflect a form of communication, the presence and (positive or negative) role of such a mechanism in inter-kingdom interaction remains to be clarified.

Besides the mechanism involving the PCA cluster, also its evolution turned out to be interesting. For plant associated fungi, like those of the genus *Trichoderma*, HGT seems to be a rather common phenomenon, especially concerning genes involved in production of secondary metabolites^89^. Hence, we tested the hypothesis that the discordance of the phylogenetic trees of the PCA cluster proteins with ascomycete phylogeny might reflect HGT of the PCA cluster. However, this was not the case – in contrast, this analysis suggested that plants had acquired part of the cluster from fungi.

Nevertheless, the similarity of the *T. harzianum* PCA cluster with that in *Metarhizium* spp^90^. suggests potential functionalities beneficial for both fungi. *Metarhizium* spp. are known endophytes^91,92^ and insect pathogens infecting hundreds of species^93,94^. In a tripartite interaction, *M. robertsii*, which comprises the PCA cluster, transfers nitrogen from insects they had infected to their plant hosts^95^. A comparable situation was shown for *Laccaria bicolor* which associates with pine and spruce and transfers nitrogen from collembola in soil to the roots it colonizes^96^. Recently, an impact of a strain belonging to *T. afroharzianum*, a species closely related to *T. harzianum*, on the interaction of a plant with pathogenic insects was shown^97,98^. Although this interaction was rather indirect via modulation of the gut microbiome, also numerous direct antagonistic effects on insects by *Trichoderma* are known^99^. Consequently, an ecological function of *Trichoderma* comparable to that of *Metarhizium* shown by Behie and colleagues^95^ warrants further investigation.

Since availability and uptake of different nitrogen sources – for example from insects, plants or soil - considerably influences secondary metabolism^100^, the upregulation of the *niiA* and *niaD* homologues upon plant recognition in *T. harzianum* B97 may not only reflect nitrogen transport to the plant, but could also be a sign for increased efforts for production of specific secondary metabolites for plant communication. Upregulation of *pca4* under these conditions would also be in line with potential nitrogen provision to the plant, especially since the pH-dependence of the catalyzed reaction under usual conditions of growth of *Trichoderma* (neutral to acidic) tends to the reverse reaction i.e. the formation of opines^101^. If indeed the coordinated up-regulation of *pca4* and *niiA*/*niaD*as a nitrogen metabolic response leads to the production of an octopine-compound, this is likely to be important for fungus-plant symbiosis as postulated by Kong et al.^102^,. Notably, although several genes associated with secondary metabolism are down-regulated, besides the strong induction of PCA cluster genes, also an NRPS encoding gene and a PKS encoding gene are slightly upregulated. These obvious shifts in secondary metabolism are in agreement with the altered pattern we observed due to the presence of the plant, but further studies are required for confirmation.

Not only chemical communication between fungi and plants represents a current subject of research, the quest for marker genes for efficient biocontrol capabilities already goes on for decades as well. This includes the question of what makes *T. harzianum* strains better than many other species of the genus. Species of the Harzianum complex are supposed to be the most common endophytic species in tropical trees^103^, with speciation leading to habitat preferences of soil or endophytism^21^. Among the *Trichoderma* spp of the Harzianum clade, which comprise the PCA cluster, there are also endophytically growing ones like *T. endophyticum* and *T. afrasin*^21^. The upregulation of the *niiA* and *niaD* homologues, which are involved in nitrogen uptake upon recognition of the plant (*nit3* and *nit6*) indicates that nitrogen metabolism also may play a role in the interaction of *T. harzianum* with the plant, although potentially also other sources of nitrogen are used than by *Metarhizium*, which degrades killed insects to deliver nitrogen to the plant^95^. The PCA cluster could at least in part explain the efficiency of *T. harzianum* in biocontrol and hence represent a genetic marker for this competence. It is likely to support beneficial communication to the plant at an early stage of colonization, which may involve pretending to be an arriving endophyte delivering additional soil/organic nitrogen. The fact that the PCA cluster is crucial for colonization by *T. harzianum* strongly indicates that the presence of this cluster in the fungus contributes to the high efficiency of members of this clade in biocontrol applications. Accordingly, microbiome analysis revealed co-occurrence of *Trichoderma* and *Metarhizium* species, both sharing the PCA cluster, in the rhizospere at high yielding field sites^104^ or with banana plants^105^.

**Fig. 6:**
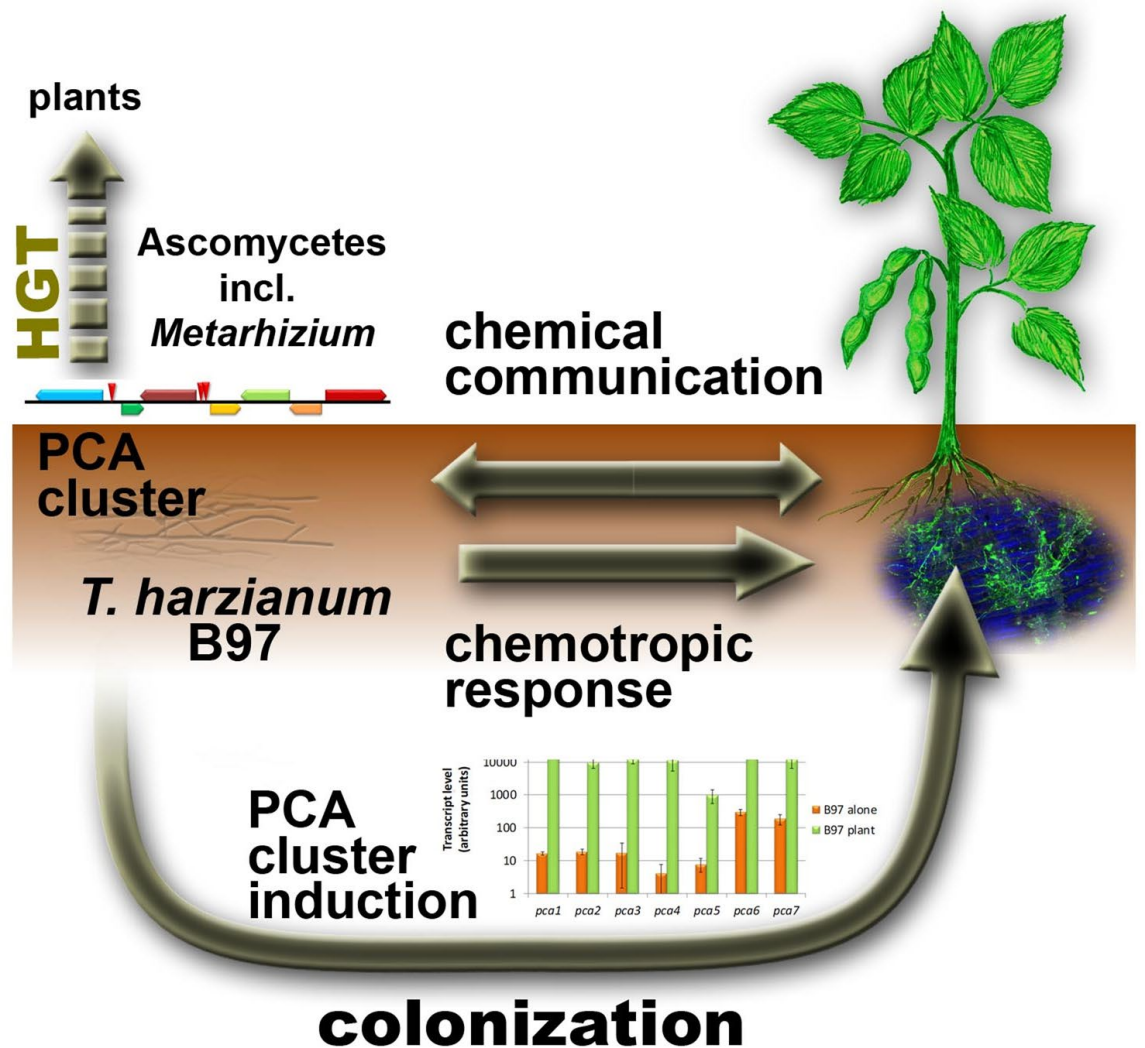
Schematic representation of the reaction of *T. harzianum* B97 to soy bean. *T. harzianum* chemotropically responds to the presence of a soy plant. Chemical communication occurs both ways due to alteration of the secondary metabolite pattern of both *T. harzianum* B97 and the soy plant. The PCA gene cluster, which highly similar to a cluster in *Metarhizium*, is strongly induced upon plant recognition and essential for effective colonization of plant roots and required for efficient colonization. Individual homologues of cluster genes were acquired by HGT by plants from ascomycetes.

In summary, we developed a strategy to simulate conditions of chemotropic response applicable for transcriptome analysis and detection of relevant secreted metabolites. Thereby, we found the PCA cluster, a putative secondary metabolite cluster essential for the very first stage of communication with a plant and enabling efficient colonization of the root surface (Fig. 6). Evolutionary analysis of the members of the cluster suggested that parts of it is not only relevant to fungi, but also to plants, which have acquired for example a *pca1* homologue from fungi by HGT. While revealing an intriguing new aspect of plant-fungus interaction, knowledge on the PCA cluster genes can also be applied to evaluate the specificity of its regulation for prediction of high efficiency biocontrol capacity during strain screening. Thereby, the presence of the cluster as well as its high induction level represent promising features for diagnostic tests in strain screening programs for novel biocontrol agents.

Additionally, future identification of the compound produced (or modified) by the genes of the PCA cluster, which facilitates colonization, may support and enhance plant-fungus interaction of diverse biocontrol agents and enable plant-protection of plant varieties with otherwise insufficient response to *Trichoderma*-based biocontrol agents. In this respect, exploration of the potential production of opin like compounds or enhancement of other nitrogen compounds beneficial for the plant will be a worthwhile research direction. Moreover, future research should also focus on identification of the transcription factor(s) or trans-acting factor(s) binding to the identified DNA-motif to facilitate detailed analysis of the regulation dynamics of the PCA cluster. Such knowledge is also likely to contribute to identification of the produced compound and to subsequent production of this chemical for possible enhancement of biocontrol agents of diverse species.

## Materials and methods

### Strains and cultivation conditions

*T. harzianum* B97^49^ was used throughout the study. For RNA analysis, the strain was revived from long term storage on malt extract agar (3% w/v). Plates containing modified Mandels Andreotti minimal medium^106^ with 0.1% (w/v) glucose as carbon source were inoculated with 10 µl of spore solution (10^8^ spores/ml) at 28 °C in constant darkness for 34 h. The modified Mandels-Andreotti medium was prepared as follows: The mineral salt solution contained 2.8 g/l (NH_4_)_2_SO_4_ (21.19 mM) (ROTH, Karlsruhe, Germany), 4.0 g/l KH_2_PO (29.39 mM) (Sigma-Aldrich, St. Louis, USA), 0.6 g/l MgSO·7H_2_O (2.43 mM) (ROTH, Karlsruhe, Germany) and 0.8 g/l CaCl_2_·2H_2_O (5.44 mM) (Merck, Darmstadt, Germany). The trace element solution contained 0.250 g/l FeSO_4_·7H_2_O (0.899 mM) (Sigma-Aldrich, St. Louis, USA), 0.085 g/l MnSO_4_·H_2_O (0.503 mM) (Merck, Darmstadt, Germany), 0.070 g/l ZnSO_4_·7H_2_O (0.243 mM) (Riedel-de Haen, Seelze, Germany) and 0.143 g/l CoCl_2_·6H_2_O (0.603 mM) (Sigma-Aldrich, St. Louis, USA) and the pH was adjusted to 2.0 with concentrated sulfuric acid. The culture medium was prepared combining 500 ml mineral salt solution, 480 ml milliQ water, 20 ml trace element solution, 0.0025% (w/v) peptone from casein (Merck, Darmstadt, Germany) to facilitate germination, 0.1% (w/v) D-glucose (ROTH, Karlsruhe, Germany) as carbon source and 1.5% (w/v) agar-agar (ROTH, Karlsruhe, Germany).

Plates were covered with cellophane in order to facilitate harvesting of mycelia. For recognition analysis, the roots of soy plants in the second leaf stage (19 days old) were washed 4 times with sterile distilled water and were placed on the plates with *T. harzianum* B97 in 3 cm distance from the growth front. After further incubation for 13 h (corresponding to the time for recognition in the chemotropic assay) in darkness, fungal mycelia were harvested for RNA isolation and agar slices from the same area were excised for evaluation of secreted metabolite production. As controls, plates with fungus but no plant and plates with plant but no fungus were used. Five plates each were pooled per sample and three biological replicates were used.

### Construction of *T. harzianum* B97 deletion strains

Vectors for deletion of *pca1* and *pca5* were constructed by yeast recombination cloning using *hph* marker constructs with 1 kb flanking regions as described previously^107^. Protoplast transformation was used for deletions in *T. harzianum* B97 parental strain with 10 mg/ml lysing enzymes (*Trichoderma harzianum*, Sigma # L-1412) and 150 µg/ml hygromycin B (Roth, Karlsruhe, Germany) for selection. Absence of the gene of interest was confirmed by PCR with primers binding inside the deleted region. In parallel, a PCR with standard primers amplifying *tef1* was performed to exclude a false negative result due to insufficient DNA quality or failed PCR assay. A list of primers used is shown in supplementary table S8 in supplementary file 2. The independent mutant strains pca1_4Ba, pca1_13Aa (lacking the *pca1* gene), pca5_27Ba and pca5_28Aa (lacking *pca5*) were used for further analyses and yielded consistent results.

### Surface sterilization of seeds and in-vitro culture of soybean plants

The soybeans (*Glycine max* (L) Merr., variety ES TENOR, Die Saat, Austria) were obtained from RWA Austria. For the surface sterilization, the soybeans were soaked in 70% ethanol for 1 min and then rinsed 3 times with sterile distilled water. Afterwards, the soybeans were transferred into a sterile beaker containing Danklorix (2.8% sodium hypochlorite (w/w), Colgate-Palmolive, Vienna, Austria) and Tween 20 (Roth, Karlsruhe, Germany) and stirred for 3 min. The soybeans were then washed five times with sterile distilled water. The excess water was removed by placing soybeans on sterile paper tissue.

For the pre-germination, the surface sterilized soybeans were placed on sterile paper tissue soaked with tap water and incubated at 26 °C and 16 h light:8 h darkness for 5 days. The seedlings were transferred to the in-vitro culture, which contained diluted Murashige & Skoog medium (to 0.5 concentration; Duchefa Biochemie, Haarlem, The Netherlands) and 0.8% (w/v) Daishin Agar (Duchefa Biochemie, Haarlem, The Netherlands) at pH 5.8. The in-vitro cultures of soybean plants were further incubated at the same condition for two more weeks.

### Preparation of plant root exudates

After surface sterilization the soybeans were planted in sterilized perlite (premium perlite 2–6, Gramoflor GmbH, Germany). The soybeans were kept in a plant culture room at 26 °C with 16 h light and 8 h darkness. After approximately 3 days of emergence, the plantlets were then allowed to grow for further 2 weeks till the second leaf stage was achieved. The plantlets were recovered from the perlite carefully and washed gently under running water to remove the perlite. Afterwards, at least 300 plantlet roots were submerged in 500 ml sterile milliQ water and kept for 2 days at room temperature. The obtained root exudates were filter sterilized through Thermo Scientific Nalgene Syringe Filter with 0.2 µM pore size and stored at −80 °C.

### Isolation of total RNA

For isolation of total RNA, mycelium of the growth front from 5 replicate plates was pooled and frozen in liquid nitrogen. Three biological replicates were used with 5 pooled plates each. Samples were then treated as described previously^108^ using the QIAGEN plant RNA kit (QIAGEN, Hilden, Germany). RNA quality and integrity were checked using Bioanalyzer 2100 (Agilent). Only high quality RNA was used for further analyses.

### Transcriptome analysis and bioinformatics

Sequencing of samples along with cDNA preparation was done in collaboration with VetCORE (Vienna, Austria) using Illumina HiSeq 50 bp single-read sequencing. The software BWA was used for mapping to the genome data of *T. harzianum* (JGI mycocosm; https://genome.jgi.doe.gov/Triha1/Triha1.home.html)^42^. Quality control of sequencing data was done using FASTQC and Trimmomatic^109^ was used for trimming of reads. HTseq was applied for read counting^110^. The software samtools was used for data processing^111^ and the limma package as implemented in R^112^ was used for determination of statistically significant differential expression (> 2fold, p-value threshold 0.01) along with the voom method^113^, quantile normalization, linear model fitting (with lmFit) and empirical Bayes methods of assessing differential expression (with eBayes^114^;. Comparison of gene expression patterns between biological replicates yielded significance scores of ≥ 0.979 for both sample sets. Sequence data are available at NCBI GEO (Gene expression omnibus) under the accession number GSE229209.

HCE3.5^115^ was applied to perform hierarchical clustering with default settings and the Poisson correlation coefficient as the similarity/distance measure. FunCat (Functional category) analysis was done with the FungiFun2 online tool^116^ based on bidirectional best hit analysis with *T. atroviride*.

### Analysis of chemotropic responses

The freshly grown spores from a 4 days old culture were recovered and suspended in 1 ml spore-solution (0.8% NaCl and 0.05% Tween 80). After separation of mycelia by centrifugation through glass wool, the spore solution was centrifuged at 8000 g for 2 min, the supernatant was discarded, and the spore pellet was resuspended in 1 ml sterile milliQ water. For the chemotropism assay, the spore solution was adjusted to 10^8^ spores per ml with sterile milliQ water. Peptone from casein (Merck, Darmstadt, Germany) was used as germination stimulator in 0.5% water agar. The concentration of peptone from casein was optimized to 0.0025% (w/v). After 13 h of incubation at 28 °C in darkness, germling orientation was monitored for at least 400 germlings per sample and chemotropic index was calculated as described earlier^32^.

### Analysis of patterns of secreted metabolites

Analysis for alteration of secondary metabolite patterns in the presence of a soy plant was essentially done as described previously^24,117^. Therefore the same conditions as applied for transcriptome analysis as outlined above were used (Fig. 1C). Application of high performance thin layer chromatography (HPTLC) and data visualization was performed as described in^24^ except that separation was done with chloroform and 1 mM trifluoroacetic acid in methanol.

### Analysis of colonization by *T. harzianum* B97 and Recombinant strains

Seeds were surface sterilized in 70% ethanol for 7 min and rinsed for 3 min with sterile milliQ water. Seeds were then put onto MEX (malt extract) plates containing either *T. harzianum* B97, two B97Δ*pca1* and two B97Δ*pca5* strains or only MEX without fungus as negative control. Seeds were then placed in sterile magenta boxes containing soil mixture (1:1:1 perlite, sand, potting soil and 25 ml of sterilized tap water), which was autoclaved twice. After 8 days at 22 °C under 12 h light:12 h darkness conditions, plants were harvested, and roots stained in 15 ml phosphate buffered saline (PBS, pH 7.2) containing 5 µg/ml wheat germ agglutinin (WGA)-AlexaFluor488 conjugate (Life Technologies, USA) and incubated for 2 h at 37 °C before rinsing three times with PBS.

Eight biological replicates were grown and treated for wild-type and controls, respectively. In case of mutant strains, each of the two independent mutants per gene-deletion was grown in 4 replicates, resulting in 8 replicate assays per mutation. Of those replicates, six were selected for microscopic analysis. On every replicate/root, several sites were monitored to ensure consistency of the results.

All observations were carried out using a confocal microscope (Olympus Fluoview FV1000 with multi-line laser FV5-LAMAR-2 and HeNe(G)laser FV10-LAHEG230-2, Japan). Observations with the confocal microscope were done at objective lenses of 10x, 20x and 40x. Between 20 and 40 X, Y, Z pictures containing 20 to 60 scans were separately taken at 405, 488, 549 nm wavelengths in blue/green/orange-red channels respectively, with the same settings each time and normal light. The image analysis software Imaris was used on the confocal microscope to visualize 3D reconstructions. X, Y, Z pictures from different channels were then merged using the Image J software (version 1.47v), and Z project stacks were then used to create the pictures as described earlier^118^.

### Phylogenetic analysis, clusters, HGT and evolution

For phylogenetic analysis, protein and nucleotide sequences were obtained from the NCBI nr database or the genome sequences available at JGI mycocosm. Sequences were aligned using Clustal X or MEGA7^119,120^ with default parameters. MEGA7 was used for phylogenetic analysis using standard parameters, the Maximum likelihood method and 1000 bootstrap cycles.

Horizontal gene transfer was analyzed using the python tool HGTphyloDetect^121^. Blastp was set to at least 1000 hits to cover close and distant relationships. Besides the conventional workflow, also the scripts for close and distant relationships implemented in HGTphyloDetect were tested in every case.

Tajima’s D was calculated using DnaSP6^122^.

Potential biosynthetic genes and clusters were analyzed using antismash^123^ as implemented in Galaxy (antismash version 6.1.1)^124^. Search for similar biosynthetic gene clusters (BGCs) was performed by CBlaster^125^. The CBlaster results for the BGCs were compared using Clinker^76^.

## Supplementary Information

Below is the link to the electronic supplementary material.

Supplementary Material 1

Supplementary Material 2

Supplementary Material 3

Supplementary Material 4

## Data Availability

All data used for this study are available in the manuscript, its supplementary files and at the NCBI GEO online repository under accession number GSE229209.

## References

[1] Harman, G. E. et al. Uses of *Trichoderma* spp. To alleviate or remediate soil and water pollution. *Adv. Appl. Microbiol.* **56**, 313–330. 10.1016/S0065-2164(04)56010-0 (2004). 15566984 10.1016/S0065-2164(04)56010-0

[2] Averill, C. et al. Defending earth’s terrestrial Microbiome. *Nat. Microbiol.*10.1038/s41564-022-01228-3 (2022). 36192539 10.1038/s41564-022-01228-3

[3] Doehlemann, G. et al. Plant pathogenic fungi. *Microbiol. Spectr.*10.1128/microbiolspec.FUNK-0023-2016 (2017). 28155813 10.1128/microbiolspec.funk-0023-2016 PMC11687436

[4] Bebber, D. P. Range-expanding pests and pathogens in a warming world. *Annu. Rev. Phytopathol.* **53**, 335–356. 10.1146/annurev-phyto-080614-120207 (2015). 26047565 10.1146/annurev-phyto-080614-120207

[5] Raza, M. M. & Bebber, D. P. Climate change and plant pathogens. *Curr. Opin. Microbiol.* **70**, 102233. 10.1016/j.mib.2022.102233 (2022). 36370642 10.1016/j.mib.2022.102233

[6] Del Buono, D. Can biostimulants be used to mitigate the effect of anthropogenic climate change on agriculture? It is time to respond. *Sci. Total Environ.* **751**, 141763. 10.1016/j.scitotenv.2020.141763 (2021). 32889471 10.1016/j.scitotenv.2020.141763

[7] Liu, X. et al. The legacy of microbial inoculants in agroecosystems and potential for tackling climate change challenges. *iScience* **25**, 103821. 10.1016/j.isci.2022.103821 (2022). 35243218 10.1016/j.isci.2022.103821 PMC8867051

[8] Khan, R. A. A. et al. Bioactive secondary metabolites from *Trichoderma* spp. against phytopathogenic bacteria and root-knot nematode. *Microorganisms*10.3390/microorganisms8030401 (2020). 32182971 10.3390/microorganisms8030401 PMC7143365

[9] Syed, A. et al. Emerging microbial biocontrol strategies for plant pathogens. *Plant. Sci.* **267**, 102–111. 10.1016/j.plantsci.2017.11.012 (2018). 29362088 10.1016/j.plantsci.2017.11.012

[10] Rangel, L. I. et al. Fungal social influencers: secondary metabolites as a platform for shaping the plant-associated community. *Plant. J.* **108**, 632–645. 10.1111/tpj.15490 (2021). 34510609 10.1111/tpj.15490

[11] Sood, M. et al. *Trichoderma*: the secrets of a multitalented biocontrol agent. *Plants (Basel)*10.3390/plants9060762 (2020). 32570799 10.3390/plants9060762 PMC7355703

[12] Ramirez-Valdespino, C. A. et al. *Trichoderma* as a model to study effector-like molecules. *Front. Microbiol.* **10**, 1030. 10.3389/fmicb.2019.01030 (2019). 31156578 10.3389/fmicb.2019.01030 PMC6529561

[13] Tyskiewicz, R. et al. *Trichoderma*: The current status of Its application in agriculture for the biocontrol of fungal phytopathogens and stimulation of plant growth. *Int. J. Mol. Sci.*10.3390/ijms23042329 (2022). 35216444 10.3390/ijms23042329 PMC8875981

[14] Schalamun, M. & Schmoll, M. *Trichoderma* – genomes and genomics as treasure troves for research towards biology, biotechnology and agriculture. *Front. Fungal Biology*10.3389/ffunb.2022.1002161 (2022). 10.3389/ffunb.2022.1002161 PMC10512326 37746224

[15] Woo, S. L. et al. *Trichoderma*: a multipurpose, plant-beneficial microorganism for eco-sustainable agriculture. *Nat. Rev. Microbiol.*10.1038/s41579-022-00819-5 (2022). 36414835 10.1038/s41579-022-00819-5

[16] Druzhinina, I. S. et al. *Trichoderma*: the genomics of opportunistic success. *Nat. Rev. Microbiol.* **9**, 749–759. 10.1038/nrmicro2637 (2011). 21921934 10.1038/nrmicro2637

[17] Harman, G. E. et al. *Trichoderma* species–opportunistic, avirulent plant symbionts. *Nat. Rev. Microbiol.* **2**, 43–56 (2004). 15035008 10.1038/nrmicro797

[18] Guzman-Guzman, P. et al. *Trichoderma* species: versatile plant symbionts. *Phytopathology* **109**, 6–16. 10.1094/PHYTO-07-18-0218-RVW (2019). 30412012 10.1094/PHYTO-07-18-0218-RVW

[19] Contreras-Cornejo, H. A. et al. Ecological functions of *Trichoderma* spp. And their secondary metabolites in the rhizosphere: interactions with plants. *FEMS Microbiol. Ecol.* **92**, fiw036. 10.1093/femsec/fiw036 (2016). 26906097 10.1093/femsec/fiw036

[20] Haueisen, J. & Stukenbrock, E. H. Life cycle specialization of filamentous pathogens - colonization and reproduction in plant tissues. *Curr. Opin. Microbiol.* **32**, 31–37. 10.1016/j.mib.2016.04.015 (2016). 27153045 10.1016/j.mib.2016.04.015

[21] Chaverri, P. et al. Systematics of the *Trichoderma Harzianum* species complex and the re-identification of commercial biocontrol strains. *Mycologia* **107**, 558–590. 10.3852/ (2015). 25661720 10.3852/14-147 PMC4885665

[22] Harman, G. E. & Uphoff, N. Symbiotic root-endophytic soil microbes improve crop productivity and provide environmental benefits. *Scientifica (Cairo)*10.1155/2019/9106395 (2019). 31065398 10.1155/2019/9106395 PMC6466867

[23] Bailey, B. & Melnick, R. *The endophytic Trichoderma* CABI International (2013).

[24] Bazafkan, H. et al. Mating type dependent partner sensing as mediated by VEL1 in *Trichoderma Reesei*. *Mol. Microbiol.* **96**, 1103–1118. 10.1111/mmi.12993 (2015). 25757597 10.1111/mmi.12993 PMC4949666

[25] Leeder, A. C. et al. The social network: Deciphering fungal Language. *Nat. Rev. Microbiol.* **9**, 440–451. 10.1038/nrmicro2580 (2011). 21572459 10.1038/nrmicro2580

[26] Macias-Rodriguez, L. et al. The interactions of *Trichoderma* at multiple trophic levels: inter-kingdom communication. *Microbiol. Res.* **240**, 126552. 10.1016/j.micres.2020.126552 (2020). 32659716 10.1016/j.micres.2020.126552

[27] Lombardi, N. et al. Root exudates of stressed plants stimulate and attract *Trichoderma* soil fungi. *Mol. Plant. Microbe Interact.*10.1094/MPMI-12-17-0310-R (2018). 29547355 10.1094/MPMI-12-17-0310-R

[28] Joo, J. H. & Hussein, K. A. Biological control and plant growth promotion properties of volatile organic Compound-producing antagonistic *Trichoderma* spp. *Front. Plant. Sci.* **13**, 897668. 10.3389/fpls.2022.897668 (2022). 35958189 10.3389/fpls.2022.897668 PMC9360753

[29] Schweiger, R. et al. Insights into metabolic changes caused by the *Trichoderma virens*-maize root interaction. *Mol. Plant. Microbe Interact.* **34**, 524–537. 10.1094/MPMI-04-20-0081-R (2021). 33166203 10.1094/MPMI-04-20-0081-R

[30] Newman, M. A. et al. MAMP (microbe-associated molecular pattern) triggered immunity in plants. *Front. Plant. Sci.* **4**, 139. 10.3389/fpls.2013.00139 (2013). 23720666 10.3389/fpls.2013.00139 PMC3655273

[31] Turra, D. & Di Pietro, A. Chemotropic sensing in fungus-plant interactions. *Curr. Opin. Plant. Biol.* **26**, 135–140. 10.1016/j.pbi.2015.07.004 (2015). 26247120 10.1016/j.pbi.2015.07.004

[32] Turra, D. et al. Fungal pathogen uses sex pheromone receptor for chemotropic sensing of host plant signals. *Nature* **527**, 521–524. 10.1038/nature15516 (2015). 26503056 10.1038/nature15516

[33] Nordzieke, D. E. et al. NADPH oxidase regulates chemotropic growth of the fungal pathogen *Fusarium oxysporum* towards the host plant. *New. Phytol*. **224**, 1600–1612. 10.1111/nph.16085 (2019). 31364172 10.1111/nph.16085

[34] Turra, D. et al. Hyphal chemotropism in fungal pathogenicity. *Semin Cell. Dev. Biol.* **57**, 69–75. 10.1016/j.semcdb.2016.04.020 (2016). 27150623 10.1016/j.semcdb.2016.04.020

[35] Stallforth, P. et al. Functional modulation of chemical mediators in microbial communities. *Trends Biochem. Sci.* **48**, 71–81. 10.1016/j.tibs.2022.07.006 (2023). 35981931 10.1016/j.tibs.2022.07.006

[36] He, D. C. et al. Biological control of plant diseases: an evolutionary and eco-economic consideration. *Pathogens*10.3390/pathogens10101311 (2021). 34684260 10.3390/pathogens10101311 PMC8541133

[37] Karlsson, M. et al. Necrotrophic mycoparasites and their genomes. *Microbiol. Spectr.*10.1128/microbiolspec.FUNK-0016-2016 (2017). 28281442 10.1128/microbiolspec.funk-0016-2016 PMC11687461

[38] Lehner, S. M. et al. Isotope-assisted screening for iron-containing metabolites reveals a high degree of diversity among known and unknown siderophores produced by trichoderma spp. *Appl. Environ. Microbiol.* **79**, 18–31. 10.1128/AEM.02339-12 (2013). 23064341 10.1128/AEM.02339-12 PMC3536107

[39] Zeilinger, S. et al. Secondary metabolism in *Trichoderma* - chemistry Meets genomics. *Fungal Biology Reviews*. **30**, 74–90. 10.1016/j.fbr.2016.05.001 (2016).

[40] Vinale, F. & Sivasithamparam, K. Beneficial effects of *Trichoderma* secondary metabolites on crops. *Phytother Res.* **34**, 2835–2842. 10.1002/ptr.6728 (2020). 32578292 10.1002/ptr.6728

[41] Shenouda, M. L. & Cox, R. J. Molecular methods unravel the biosynthetic potential of *Trichoderma* species. *RSC Adv.* **11**, 3622–3635. 10.1039/d0ra09627j (2021). 35424278 10.1039/d0ra09627j PMC8694227

[42] Druzhinina, I. S. et al. Massive lateral transfer of genes encoding plant cell wall-degrading enzymes to the mycoparasitic fungus *Trichoderma* from its plant-associated hosts. *PLoS Genet.* **14**, e1007322. 10.1371/journal.pgen.1007322 (2018). 29630596 10.1371/journal.pgen.1007322 PMC5908196

[43] Druzhinina, I. S. et al. Several steps of lateral gene transfer followed by events of ‘birth-and-death’ evolution shaped a fungal sorbicillinoid biosynthetic gene cluster. *BMC Evol. Biol.* **16**, 269. 10.1186/s12862-016-0834-6 (2016). 28010735 10.1186/s12862-016-0834-6 PMC5182515

[44] Soucy, S. M. et al. Horizontal gene transfer: Building the web of life. *Nat. Rev. Genet.* **16**, 472–482. 10.1038/nrg3962 (2015). 26184597 10.1038/nrg3962

[45] Huang, J. Horizontal gene transfer in eukaryotes: the weak-link model. *Bioessays* **35**, 868–875. 10.1002/bies.201300007 (2013). 24037739 10.1002/bies.201300007 PMC4033532

[46] Leger, M. M. et al. Demystifying eukaryote lateral gene transfer (Response to Martin 2017 DOI: 10.1002/bies.201700115). *Bioessays* **40**, e1700242. 10.1002/bies.201700242 (2018). 29543982 10.1002/bies.201700242

[47] Martin, W. F. Too much eukaryote LGT. *Bioessays*10.1002/bies.201700115 (2017). 29068466 10.1002/bies.201700115

[48] Danchin, E. G. Lateral gene transfer in eukaryotes: tip of the iceberg or of the ice cube? *BMC Biol.* **14**, 101. 10.1186/s12915-016-0330-x (2016). 27863503 10.1186/s12915-016-0330-x PMC5116166

[49] Compant, S. et al. Draft genome sequence of the root-colonizing fungus Trichoderma harzianum B97. *Genome Announc*10.1128/genomeA.00137-17 (2017). 28360171 10.1128/genomeA.00137-17 PMC5374245

[50] Lareen, A. et al. Plant root-microbe communication in shaping root microbiomes. *Plant. Mol. Biol.* **90**, 575–587. 10.1007/s11103-015-0417-8 (2016). 26729479 10.1007/s11103-015-0417-8 PMC4819777

[51] Brownlee, A. G. & Arst, H. N. Jr. Nitrate uptake in *Aspergillus Nidulans* and involvement of the third gene of the nitrate assimilation gene cluster. *J. Bacteriol.* **155**, 1138–1146 (1983). 6350263 10.1128/jb.155.3.1138-1146.1983 PMC217809

[52] Johnstone, I. L. et al. Isolation and characterisation of the *crnA-niiA-niaD* gene cluster for nitrate assimilation in *Aspergillus Nidulans*. *Gene* **90**, 181–192 (1990). 2205530 10.1016/0378-1119(90)90178-t

[53] Gao, R. et al. The evolutionary and functional paradox of cerato-platanins in fungi. *Appl. Environ. Microbiol.*10.1128/AEM.00696-20 (2020). 32332135 10.1128/AEM.00696-20 PMC7301859

[54] Gomes, E. V. et al. The cerato-platanin protein Epl-1 from *Trichoderma Harzianum* is involved in mycoparasitism, plant resistance induction and self cell wall protection. *Sci. Rep.* **5**, 17998. 10.1038/srep17998 (2015). 26647876 10.1038/srep17998 PMC4673615

[55] Gomes, E. V. et al. Involvement of *Trichoderma Harzianum* Epl-1 protein in the regulation of *Botrytis* virulence- and tomato defense-related genes. *Front. Plant. Sci.* **8**, 880. 10.3389/fpls.2017.00880 (2017). 28611802 10.3389/fpls.2017.00880 PMC5446994

[56] Szklarczyk, D. et al. The STRING database in 2021: customizable protein-protein networks, and functional characterization of user-uploaded gene/measurement sets. *Nucleic Acids Res.* **49**, D605–D612. 10.1093/nar/gkaa1074 (2021). 33237311 10.1093/nar/gkaa1074 PMC7779004

[57] Lind, A. L. et al. Examining the evolution of the regulatory circuit controlling secondary metabolism and development in the fungal genus *Aspergillus*. *PLoS Genet.* **11**, e1005096. 10.1371/journal.pgen.1005096 (2015). 25786130 10.1371/journal.pgen.1005096 PMC4364702

[58] Kurucz, V. et al. Additional oxidative stress reroutes the global response of *Aspergillus fumigatus* to iron depletion. *BMC Genom.* **19**, 357. 10.1186/s12864-018-4730-x (2018). 10.1186/s12864-018-4730-x PMC5946477 29747589

[59] Losada, L. et al. Large-scale transcriptional response to hypoxia in *Aspergillus fumigatus* observed using RNAseq identifies a novel hypoxia regulated NcRNA. *Mycopathologia* **178**, 331–339. 10.1007/s11046-014-9779-8 (2014). 24996522 10.1007/s11046-014-9779-8 PMC4239182

[60] Kimura, M. et al. Trichothecene 3-O-acetyltransferase protects both the producing organism and transformed yeast from related mycotoxins. Cloning and characterization of Tri101. *J. Biol. Chem.* **273**, 1654–1661. 10.1074/jbc.273.3.1654 (1998). 9430709 10.1074/jbc.273.3.1654

[61] Cardoza, R. E. et al. Identification of loci and functional characterization of trichothecene biosynthesis genes in filamentous fungi of the genus *Trichoderma*. *Appl. Environ. Microbiol.* **77**, 4867–4877 (2011). 21642405 10.1128/AEM.00595-11 PMC3147405

[62] Shentu, X. et al. Tri11, tri3, and tri4 genes are required for trichodermin biosynthesis of trichoderma Brevicompactum. *AMB Express*. **8**, 58. 10.1186/s13568-018-0585-4 (2018). 29667033 10.1186/s13568-018-0585-4 PMC5904096

[63] Degenkolb, T. et al. The *Trichoderma Brevicompactum* clade: a separate lineage with new species, new peptaibiotics, and Mycotoxins. *Mycological Progress*. **7**, 177–219. 10.1007/s11557-008-0563-3 (2008).

[64] Lamdan, N. L. et al. Secretome of *Trichoderma* interacting with maize roots: role in induced systemic resistance. *Mol. Cell. Proteom.* **14**, 1054–1063. 10.1074/mcp.M114.046607 (2015). 10.1074/mcp.M114.046607 PMC4390251 25681119

[65] Malinich, E. A. et al. Differential expression analysis of *Trichoderma virens* RNA reveals a dynamic transcriptome during colonization of *Zea Mays* roots. *BMC Genom.* **20**, 280. 10.1186/s12864-019-5651-z (2019). 10.1186/s12864-019-5651-z PMC6458689 30971198

[66] Moran-Diez, M. E. et al. Host-specific transcriptomic pattern of *Trichoderma virens* during interaction with maize or tomato roots. *BMC Genom.* **16**, 8. 10.1186/s12864-014-1208-3 (2015). 10.1186/s12864-014-1208-3 PMC4326404 25608961

[67] Nogueira-Lopez, G. et al. The apoplastic secretome of *Trichoderma virens* during interaction with maize roots shows an Inhibition of plant defence and scavenging oxidative stress secreted proteins. *Front. Plant. Sci.* **9**, 409. 10.3389/fpls.2018.00409 (2018). 29675028 10.3389/fpls.2018.00409 PMC5896443

[68] Rubio, M. B. et al. Comparative study of *Trichoderma* gene expression in interactions with tomato plants using high-density oligonucleotide microarrays. *Microbiology* **158**, 119–128. 10.1099/mic.0.052118-0 (2012). 22075029 10.1099/mic.0.052118-0

[69] Atanasova, L. et al. Two hundred *Trichoderma* species recognized on the basis of molecular phylogeny. In *Trichoderma - Biology and Applications* (eds Mukherjee, P. K. et al.) 10–42 (CAB International, 2013).

[70] Grigoriev, I. V. et al. MycoCosm portal: gearing up for 1000 fungal genomes. *Nucleic Acids Res.* **42**, D699–704. 10.1093/nar/gkt1183 (2014). 24297253 10.1093/nar/gkt1183 PMC3965089

[71] de Man, T. J. et al. Small genome of the fungus *Escovopsis weberi*, a specialized disease agent of ant agriculture. *Proc. Natl. Acad. Sci. U S A*. **113**, 3567–3572. 10.1073/pnas.1518501113 (2016). 26976598 10.1073/pnas.1518501113 PMC4822581

[72] Wang, X. et al. Genomic and transcriptomic analysis of the endophytic fungus *Pestalotiopsis fici* reveals its lifestyle and high potential for synthesis of natural products. *BMC Genom.* **16**, 28. 10.1186/s12864-014-1190-9 (2015). 10.1186/s12864-014-1190-9 PMC4320822 25623211

[73] Schafhauser, T. et al. Draft genome sequence of *Talaromyces Islandicus* (*Penicillium islandicum*) WF-38-12, a neglected mold with significant biotechnological potential. *J. Biotechnol.* **211**, 101–102. 10.1016/j.jbiotec.2015.07.004 (2015). 26197417 10.1016/j.jbiotec.2015.07.004

[74] Sbaraini, N. et al. Secondary metabolite gene clusters in the entomopathogen fungus *Metarhizium anisopliae*: genome identification and patterns of expression in a cuticle infection model. *BMC Genom.* **17**, 736. 10.1186/s12864-016-3067-6 (2016). 10.1186/s12864-016-3067-6 PMC5088523 27801295

[75] Donzelli, B. G. & Krasnoff, S. B. Molecular genetics of secondary chemistry in *Metarhizium* fungi. *Adv. Genet.* **94**, 365–436. 10.1016/bs.adgen.2016.01.005 (2016). 27131330 10.1016/bs.adgen.2016.01.005

[76] Gilchrist, C. L. M. et al. Cblaster: a remote search tool for rapid identification and visualization of homologous gene clusters. *Bioinform Adv.* **1**, vbab016. 10.1093/bioadv/vbab016 (2021). 36700093 10.1093/bioadv/vbab016 PMC9710679

[77] Gilchrist, C. L. M. & Chooi, Y. H. Clinker & clustermap.js: automatic generation of gene cluster comparison figures. *Bioinformatics* **37**, 2473–2475. 10.1093/bioinformatics/btab007 (2021). 33459763 10.1093/bioinformatics/btab007

[78] Rolfe, S. A. et al. Crying out for help with root exudates: adaptive mechanisms by which stressed plants assemble health-promoting soil microbiomes. *Curr. Opin. Microbiol.* **49**, 73–82. 10.1016/j.mib.2019.10.003 (2019). 31731229 10.1016/j.mib.2019.10.003

[79] Harman, G. et al. Benefits to plant health and productivity from enhancing plant microbial symbionts. *Front. Plant. Sci.* **11**, 610065. 10.3389/fpls.2020.610065 (2020). 33912198 10.3389/fpls.2020.610065 PMC8072474

[80] Hanson, J. R. The chemistry of the bio-control agent, *Trichoderma Harzianum*. *Sci. Prog*. **88**, 237–248. 10.3184/003685005783238372 (2005). 16961094 10.3184/003685005783238372 PMC10367512

[81] Guo, R. et al. Structures and biological activities of secondary metabolites from *Trichoderma harzianum*. *Mar. Drugs*10.3390/md20110701 (2022). 36355024 10.3390/md20110701 PMC9696559

[82] Keswani, C. et al. Unraveling the efficient applications of secondary metabolites of various *Trichoderma* spp. *Appl. Microbiol. Biotechnol.* **98**, 533–544. 10.1007/s00253-013-5344-5 (2014). 24276619 10.1007/s00253-013-5344-5

[83] Villalobos-Escobedo, J. M. et al. The fungal NADPH oxidase is an essential element for the molecular dialog between *Trichoderma* and *Arabidopsis*. *Plant. J.* **103**, 2178–2192. 10.1111/tpj.14891 (2020). 32578269 10.1111/tpj.14891

[84] Subramoni, S. et al. *Agrobacterium tumefaciens* responses to plant-derived signaling molecules. *Front. Plant. Sci.* **5**, 322. 10.3389/fpls.2014.00322 (2014). 25071805 10.3389/fpls.2014.00322 PMC4086400

[85] Beauchamp, C. J. et al. Fungal catabolism of crown gall opines. *Appl. Environ. Microbiol.* **56**, 150–155. 10.1128/aem.56.1.150-155.1990 (1990). 16348087 10.1128/aem.56.1.150-155.1990 PMC183264

[86] Morey, J. R. & Kehl-Fie, T. E. Bioinformatic mapping of opine-like zincophore biosynthesis in bacteria. *mSystems. *10.1128/mSystems.00554-20. 10.1128/mSystems.00554-20 PMC7438024 32817386

[87] McFarlane, J. S. et al. Staphylopine and Pseudopaline dehydrogenase from bacterial pathogens catalyze reversible reactions and produce stereospecific metallophores. *J. Biol. Chem.* **294**, 17988–18001. 10.1074/jbc.RA119.011059 (2019). 31615895 10.1074/jbc.RA119.011059 PMC6879343

[88] Song, L. et al. Mechanistic insights into staphylopine-mediated metal acquisition. *Proc. Natl. Acad. Sci. U S A*. **115**, 3942–3947. 10.1073/pnas.1718382115 (2018). 29581261 10.1073/pnas.1718382115 PMC5899449

[89] Spatafora, J. W. & Bushley, K. E. Phylogenomics and evolution of secondary metabolism in plant-associated fungi. *Curr. Opin. Plant. Biol.* **26**, 37–44. 10.1016/j.pbi.2015.05.030 (2015). 26116974 10.1016/j.pbi.2015.05.030

[90] Sheng, H. et al. *Metarhizium*: an opportunistic middleman for multitrophic lifestyles. *Curr. Opin. Microbiol.* **69**, 102176. 10.1016/j.mib.2022.102176 (2022). 35872503 10.1016/j.mib.2022.102176

[91] Sasan, R. K. & Bidochka, M. J. The insect-pathogenic fungus *Metarhizium Robertsii* (Clavicipitaceae) is also an endophyte that stimulates plant root development. *Am. J. Bot.* **99**, 101–107. 10.3732/ajb.1100136 (2012). 22174335 10.3732/ajb.1100136

[92] Wyrebek, M. et al. Three sympatrically occurring species of *Metarhizium* show plant rhizosphere specificity. *Microbiol. (Reading)*. **157**, 2904–2911. 10.1099/mic.0.051102-0 (2011). 10.1099/mic.0.051102-0 21778205

[93] Roberts, D. W. & Hajek, A. E. Entomopathogenic fungi as bioinsecticides. In *Frontiers in Industrial Mycology* 144–159 (Springer, 1992).

[94] St Leger, R. J. Biology and mechanisms of insect cuticle invation by Deuteromycete fungal pathogens. In *Parasites and pathogens of insects* (eds Beckage, N. E. et al.) 211–229 (Academic Press Inc, 1993).

[95] Behie, S. W. et al. Endophytic insect-parasitic fungi translocate nitrogen directly from insects to plants. *Science* **336**, 1576–1577. 10.1126/science.1222289 (2012). 22723421 10.1126/science.1222289

[96] Klironomos, J. N. & Hart, M. M. Food-web dynamics. Animal nitrogen swap for plant carbon. *Nature* **410**, 651–652. 10.1038/35070643 (2001). 11287942 10.1038/35070643

[97] Di Lelio, I. et al. A soil fungus confers plant resistance against a phytophagous insect by disrupting the symbiotic role of its gut microbiota. *Proc. Natl. Acad. Sci. U S A*. **120**, e2216922120. 10.1073/pnas.2216922120 (2023). 36848561 10.1073/pnas.2216922120 PMC10013743

[98] Monte, E. The sophisticated evolution of *Trichoderma* to control insect pests. *Proc. Natl. Acad. Sci. U S A*. **120**, e2301971120. 10.1073/pnas.2301971120 (2023). 36913591 10.1073/pnas.2301971120 PMC10041122

[99] Poveda, J. *Trichoderma* as biocontrol agent against pests: new uses for a mycoparasite. *Biol. Control*. **159**, 104634. 10.1016/j.biocontrol.2021.104634 (2021).

[100] Tudzynski, B. Nitrogen regulation of fungal secondary metabolism in fungi. *Front. Microbiol.* **5**, 656. 10.3389/fmicb.2014.00656 (2014). 25506342 10.3389/fmicb.2014.00656 PMC4246892

[101] Müller, A. et al. Putative reaction mechanism of heterologously expressed octopine dehydrogenase from the great scallop, *Pecten Maximus* (L). *FEBS J.* **274**, 6329–6339. 10.1111/j.1742-4658.2007.06151.x (2007). 18028427 10.1111/j.1742-4658.2007.06151.x

[102] Kong, D. et al. Fungus-derived opine enhances plant photosynthesis. *J. Adv. Res.* **75**, 65–77. 10.1016/j.jare.2024.11.029 (2025). 39592078 10.1016/j.jare.2024.11.029 PMC12536604

[103] Chaverri, P. et al. *Trichoderma amazonicum*, a new endophytic species on *Hevea Brasiliensis* and *H. guianensis* from the Amazon basin. *Mycologia* **103**, 139–151 (2011). 20943534 10.3852/10-078

[104] Bandara, A. Y. et al. Soybean roots and soil from high- and low-yielding field sites have different Microbiome composition. *Front. Microbiol.* **12**, 675352. 10.3389/fmicb.2021.675352 (2021). 34917042 10.3389/fmicb.2021.675352 PMC8669749

[105] Ciancio, A. et al. Rhizosphere 16S-ITS metabarcoding profiles in banana crops are affected by nematodes, cultivation, and local Climatic variations. *Front. Microbiol.* **13**, 855110. 10.3389/fmicb.2022.855110 (2022). 35756021 10.3389/fmicb.2022.855110 PMC9218937

[106] Mandels, M. & Andreotti, R. Problems and challenges in the cellulose to cellulase fermentation. *Proc. Biochem.* **13**, 6–13 (1978).

[107] Schuster, A. et al. A versatile toolkit for high throughput functional genomics with *Trichoderma Reesei*. *Biotechnol. Biofuels*. **5**, 1. 10.1186/1754-6834-5-1 (2012). 22212435 10.1186/1754-6834-5-1 PMC3260098

[108] Tisch, D. et al. New insights into the mechanism of light modulated signaling by heterotrimeric G-proteins: ENVOY acts on *gna1* and *gna3* and adjusts cAMP levels in *Trichoderma Reesei* (*Hypocrea jecorina*). *Fungal Genet. Biol.* **48**, 631–640. 10.1016/j.fgb.2010.12.009 (2011). 21220037 10.1016/j.fgb.2010.12.009 PMC3082050

[109] Keel, B. N. & Snelling, W. M. Comparison of Burrows-Wheeler Transform-Based mapping algorithms used in High-Throughput Whole-Genome sequencing: application to illumina data for livestock genomes. *Front. Genet.* **9**, 35. 10.3389/fgene.2018.00035 (2018). 29535759 10.3389/fgene.2018.00035 PMC5834436

[110] Bolger, A. M. et al. Trimmomatic: a flexible trimmer for illumina sequence data. *Bioinformatics* **30**, 2114–2120. 10.1093/bioinformatics/btu170 (2014). 24695404 10.1093/bioinformatics/btu170 PMC4103590

[111] Anders, S. et al. HTSeq–a python framework to work with high-throughput sequencing data. *Bioinformatics* **31**, 166–169. 10.1093/bioinformatics/btu638 (2015). 25260700 10.1093/bioinformatics/btu638 PMC4287950

[112] Li, H. et al. The sequence Alignment/Map format and samtools. *Bioinformatics* **25**, 2078–2079. 10.1093/bioinformatics/btp352 (2009). 19505943 10.1093/bioinformatics/btp352 PMC2723002

[113] Ritchie, M. E. et al. Limma powers differential expression analyses for RNA-sequencing and microarray studies. *Nucleic Acids Res.* **43**, e47. 10.1093/nar/gkv007 (2015). 25605792 10.1093/nar/gkv007 PMC4402510

[114] Law, C. W. et al. Voom: precision weights unlock linear model analysis tools for RNA-seq read counts. *Genome Biol.* **15**, R29. 10.1186/gb-2014-15-2-r29 (2014). 24485249 10.1186/gb-2014-15-2-r29 PMC4053721

[115] Smyth, G. K. Linear models and empirical bayes methods for assessing differential expression in microarray experiments. *Stat. Appl. Genet. Mol. Biol.*10.2202/1544-6115.1027 (2004). 16646809 10.2202/1544-6115.1027

[116] Seo, J. et al. An interactive power analysis tool for microarray hypothesis testing and generation. *Bioinformatics* **22**, 808–814. 10.1093/bioinformatics/btk052 (2006). 16418236 10.1093/bioinformatics/btk052

[117] Priebe, S. et al. FungiFun2: a comprehensive online resource for systematic analysis of gene lists from fungal species. *Bioinformatics* **31**, 445–446. 10.1093/bioinformatics/btu627 (2015). 25294921 10.1093/bioinformatics/btu627 PMC4308660

[118] Hinterdobler, W. et al. The role of PKAc1 in gene regulation and trichodimerol production in trichoderma Reesei. *Fungal Biol. Biotechnol.* **6**, 12. 10.1186/s40694-019-0075-8 (2019). 31528353 10.1186/s40694-019-0075-8 PMC6734591

[119] Pierron, R. et al. Deciphering the niches of colonisation of *Vitis vinifera* L. by the Esca-Associated fungus *Phaeoacremonium aleophilum* using a Gfp marked strain and cutting systems. *PLoS One*. **10**, e0126851. 10.1371/journal.pone.0126851 (2015). 26061034 10.1371/journal.pone.0126851 PMC4464894

[120] Kumar, S. et al. MEGA7: molecular evolutionary eenetics analysis version 7.0 for bigger datasets. *Mol. Biol. Evol.* **33**, 1870–1874. 10.1093/molbev/msw054 (2016). 27004904 10.1093/molbev/msw054 PMC8210823

[121] Thompson, J. D. et al. The CLUSTAL_X windows interface: flexible strategies for multiple sequence alignment aided by quality analysis tools. *Nucleic Acids Res.* **25**, 4876–4882 (1997). 9396791 10.1093/nar/25.24.4876 PMC147148

[122] Yuan, L. et al. HGTphyloDetect: facilitating the identification and phylogenetic analysis of horizontal gene transfer. *Brief. Bioinform*10.1093/bib/bbad035 (2023). 36752380 10.1093/bib/bbad035 PMC10025432

[123] Rozas, J. et al. DnaSP 6: DNA sequence polymorphism analysis of large data sets. *Mol. Biol. Evol.* **34**, 3299–3302. 10.1093/molbev/msx248 (2017). 29029172 10.1093/molbev/msx248

[124] Blin, K. et al. AntiSMASH 6.0: improving cluster detection and comparison capabilities. *Nucleic Acids Res.* **49**, W29–W35. 10.1093/nar/gkab335 (2021). 33978755 10.1093/nar/gkab335 PMC8262755

[125] The et al et al. The galaxy platform for accessible, reproducible and collaborative biomedical analyses: 2022 update. *Nucleic Acids Res.* **50**, W345–W351. 10.1093/nar/gkac247 (2022). 35446428 10.1093/nar/gkac247 PMC9252830

