# Supplementary figures and images for: Plant recognition by Trichoderma Harzianum elicits upregulation of a novel secondary metabolite cluster required for colonization
Source: Sci Rep. 2026 Jan 20;16:3945. doi: 10.1038/s41598-025-33935-2 (PMC12855858; doi:10.1038/s41598-025-33935-2)

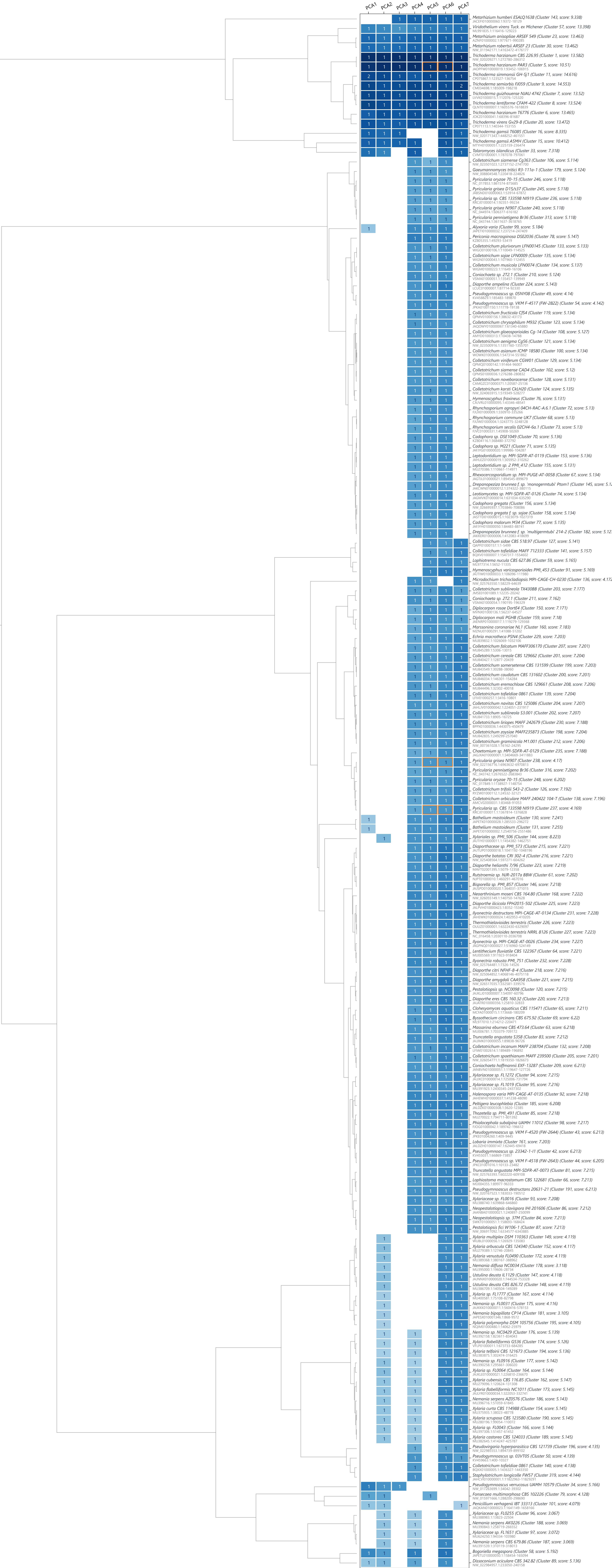

Supplement: Supplementary file 1 — Supplementary Material 1 [file 41598_2025_33935_MOESM1_ESM.pdf]
