# Supplementary material for: Plant recognition by Trichoderma Harzianum elicits upregulation of a novel secondary metabolite cluster required for colonization
Source: Sci Rep. 2026 Jan 20;16:3945. doi: 10.1038/s41598-025-33935-2 (PMC12855858; doi:10.1038/s41598-025-33935-2)
Supplement: Supplementary file 3 — Supplementary Material 3 [file 41598_2025_33935_MOESM3_ESM.pdf]

Plant recognition by *Trichoderma harzianum* elicits upregulation of a novel secondary metabolite cluster required for colonization

Miriam Schalamun[1], Guofen Li[1], Wolfgang Hinterdobler[1,§], Dominik K. Großkinsky[1], Stephane Compant[1], Assia Dreux-Zigha[2] , Jennifer Gerke[3], Russell Cox[3] and Monika Schmoll[1,4]*

[1]*AIT Austrian Institute of Technology GmbH, Center for Health and Bioresources, Konrad Lorenz Strasse 24, 3430 Tulln, Austria*
[2] *Greencell, 63360 St Beauzire, France*
[3] *Leibnitz University Hannover, Institute of Organic Chemistry, Schneiderweg 38, 30167 Hannover, Germany*
[4] *University of Vienna, Department of Microbiology and Ecosystem Science, Division of Terrestrial Ecosystem Research, Djerassiplatz 1, 1030 Vienna, Austria*

Supplementary material

Table of contents

Supplementary figure S1 – Biological replicates of fungal plant interaction (HPTLC) 2

Supplementary data 1 **3**

Regulation of the PCA-cluster genes and their homologues in T. harzianum *and* T. virens 3

supplementary table S1A 3
supplemantary table S1B 3
supplementary table S2 4
supplementary table S3 4
supplementary table S4A 4
supplementary table S4B 5

Supplementary data 2 **6**

Indications that individual genes of the PCA cluster presumably originate from plants and bacteria 6

Figure S2 7

The PCA cluster was not acquired by horizontal gene transfer 8

supplementary table S5 9

The PCA cluster is largely subject to balancing evolution 10

supplementary table S6 10

Supplementary table S7 – Blastp analysis of PCA cluster genes 10
Supplementary table S8 – Oligonucleotides used in the study 11

References **12**

Supplementary figure S1 – Biological replicates of fungal plant interaction (HPTLC)

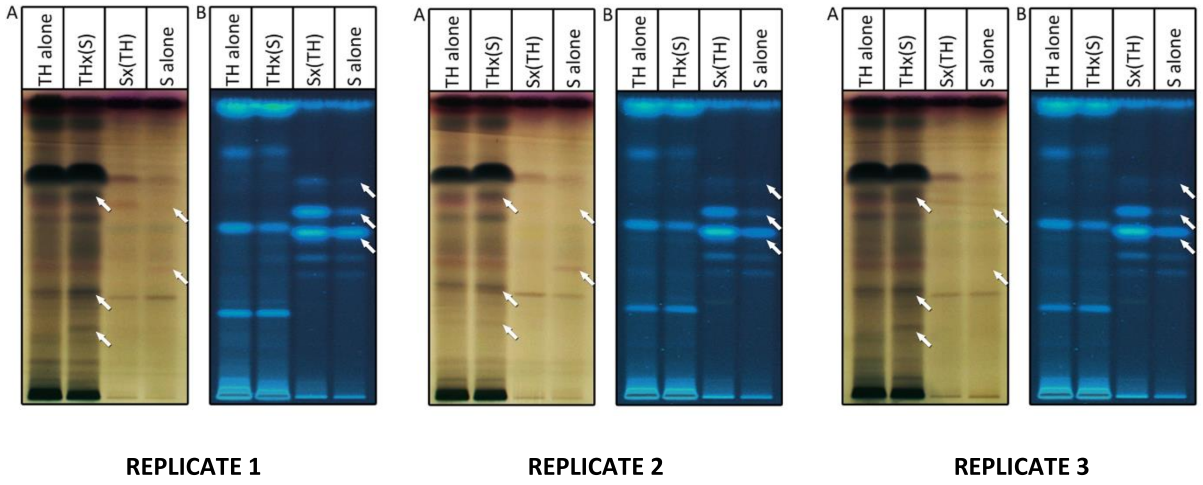

Supplementary figure S1. **Assessment of *T. harzianum* B97 – plant interaction**. HPTLC analysis of *T. harzianum* B97 alone on the plate (TH alone), *T. harzianum* B97 in the presence of the root of soy plant (THx(S)), the root of soy plant in the presence of the fungus (Sx(TH)) and the root of soy plant alone (S alone). Two different visualizations are provided (A: Visible light after anisaldehyde derivatization and B: Remission at 366 nm) and show differentially secreted metabolites between interaction partners alone and in combination. Arrows mark bands with different abundance in the presence or absence of interaction.

Three different, independent biological replicates are shown.

Supplementary data 1

Regulation of the PCA-cluster genes and their homologues in T. virens

Considering the extreme induction of the PCA-cluster genes upon plant recognition, we were interested whether these genes are regulated during direct interaction with the plant as well. To get a broad picture on regulation, we re-analyzed different assays of plant interaction using GEO2R [1], which enables re-analysis and applies rigorous statistics on datasets submitted to NCBI GEO. For *T. virens*, which has the comlete PCA-cluster in its genome, we found in an RNAseq study that none of the pca genes was regulated significantly (p-value< 0.05, >2fold) upon interaction with tomato roots or maize roots (GSE64344; [2]) (supplementary tables S1A and S1B).

Supplementary table S1A. Transcript regulation of homologues of PCA-cluster genes in *T. virens* upon interaction with maize

Moran-Diez et al., 2015
***Trichoderma virens* interacting with maize**

pca-gene	sequence ID of homologue	p-value	logFC	SPOT_ID
pca1	7697	2.32e-01	2.1869773	GT_Tvirens_Specific_59978
pca2	10272	4.74e-01	1.4945972	GT_Tvirens_Specific_53545
pca3	13793	6.01e-01	1.1632896	GT_Tvirens_Specific_70952
pca3	11715	6.19e-01	1.2200825	GT_Tvirens_Specific_70952
pca4	7308	5.68e-01	1.2082777	GT_Tvirens_Specific_53537
pca5	9046	1.94e-01	2.6819628	GT_Tvirens_Specific_53528
pca6	1565	6.70e-01	0.7939574	GT_Tvirens_Specific_53536
pca7	3418	5.18e-01	1.4426072	GT_Tvirens_Specific_59982

Supplementary table S1B. Transcript regulation of homologues of PCA-cluster genes in *T. virens* upon interaction with tomato.

Moran-Diez et al., 2015
***Trichoderma virens* interacting with tomato**

pca-gene	sequence ID of homologue	p-value	logFC	SPOT_ID
pca1	7697	2.17e-02	4.563713	GT_Tvirens_Specific_59978
pca2	10272	1.48e-01	3.099173	GT_Tvirens_Specific_53545
pca3	13793	9.62e-02	3.910878	GT_Tvirens_Specific_70952
pca4	7308	3.39e-02	4.959625	GT_Tvirens_Specific_53537
pca5	9046	1.65e-01	2.859747	GT_Tvirens_Specific_53528
pca6	1565	8.68e-02	3.371512	GT_Tvirens_Specific_53536
pca7	3418	1.06e-01	3.817166	GT_Tvirens_Specific_59982

In a further RNAseq study [3], where a hydroponic system system was used, only *pca2, pca4, pca5* and *pca7* are represented. There, plant roots reaching into a nutrient solution were combined with pre-cultivated mycelia and shaken with 50 rpm. In contrast, our assay is based on solid media, in which diffusion is more important and volatile organic compounds (VOCs) may contribute to the interaction. The transcription levels of the detected genes remained below the threshold of 2fold significant regulation (p-value threshold <0.05) in the early so-called „recognition“ phase 6 hours after addition of mycelia to plant roots. Thirty hours after inoculation, all four genes are downregulated 7 – 20 fold compared to mycelium in the absence of plant roots (supplementary table S2).

Supplementary table S2. Transcript regulation of homologues of PCA-cluster genes in *T. virens* upon interaction with maize.

Malinich et al., 2019
***Trichoderma virens* interacting with maize roots**

		recognition phase (6 hours post inoculation)		colonization phase (30 hours post inoculation)	
**pca-gene**	**sequence ID of homologue**	**p-value**	**logFC**	**p-value**	**logFC**
pca2	**53545**	**0,552784458**	**-0,287459231**	**0,000161232**	**-3,329906741**
pca4	**53537**	**0,076522489**	**-0,735189859**	**0,000182013**	**-4,324763207**
pca5	**53536**	**0,000387048**	**-0,855144785**	**1,50E-07**	**-3,779271537**
pca7	**59982**	**0,527697499**	**-0,283252055**	**0,002780259**	**-2,871065074**

Earlier studies relied on the EST-dataset of the TrichoEST project, which comprised 12662 unique genes originating from 8 *Trichoderma* spp (including *T. harzianum*) cultivated under diverse conditions [4]. Screening of these sequences revealed that only *pca6* was represented among the sequences of *T. harzianum* and closely related sequences to *pca3* were present in *T. aggressivum* (supplementary table S3). This low representation supports the hypothesis that the PCA cluster is not relevant for axenic growth under diverse nutrient conditions and – limitations.

Supplementary table S3. Representation of PCA cluster genes among the TrichoEST clones

TrichoEST hits to *Trichoderma harzianum* PCA cluster genes

**pca-gene**	**GenBank accession of EST**	**EST from species**	**Triha1 ID (JGI)**
pca6	AJ914873.1	*T. harzianum*	513505
pca6	AJ915042.1	*T. harzianum*	513505
pca6	AJ914239.1	*T. harzianum*	513505
pca3	AJ911526.1	*T. aggressivum*	513502
pca3	AJ910300.1	*T. aggressivum*	513502
pca6	AJ917970.1	*T. stromaticum*	513505
pca6	AJ918137.1	*T. stromaticum*	513505

pca1, pca2, pca4, pca5 and *pca7* are not represented in TrichoEST sequences

In a study based on these ESTs along with the whole genome of *T. virens*, interaction of *T. harzianum* and *T. virens* was analyzed for their transcriptomic response to tomato roots [5]. However, neither the *T. virens* sequences nor the *T. harzianum* sequences provided any indication of a regulation of the PCA-cluster genes during plant interaction (GSE29171; [5]) (supplementary table S4A, S4B).

Supplementary table S4A. Transcript regulation of homologues of PCA-cluster genes in *T. harzianum* upon interaction with tomato.

***Trichoderma virens* interacting with tomato**

**pca-gene**	**sequence ID**	**p-value**	**logFC**	**SPOT_ID**
pca1	TRIVE_59978	2.79e-03	6.57e-01	jgi|Trive1|59978|fgenesh1_pm.C_scaffold_56000001
pca2	TRIVE_53545	5.75e-01	1.00e-01	jgi|Trive1|53545|e_gw1.56.8.1
pca3	TRIVE_70952	3.99e-02	3.63e-01	jgi|Trive1|70952|fgenesh1_pg.C_scaffold_56000003
pca4	TRIVE_53537	8.02e-04	7.46e-01	jgi|Trive1|53537|e_gw1.56.24.1
pca5	TRIVE_53528	1.91e-02	5.60e-01	jgi|Trive1|53528|e_gw1.56.11.1
pca6	TRIVE_53536	2.16e-04	9.82e-01	jgi|Trive1|53536|e_gw1.56.29.1
pca7	TRIVE_59982	5.71e-04	7.59e-01	jgi|Trive1|59982|fgenesh1_pm.C_scaffold_56000005

Supplementary table S4B. Transcript regulation of homologues of PCA-cluster genes in *T. virens* upon interaction with tomato.

***Trichderma harzianum* interacing with tomato**

pca-gene	sequence ID	p-value	logFC	SPOT_ID	GenBank accession of EST
pca1	TRIVE_59978	6.08e-01	-0.12889578	jgi|Trive1|59978|fgenesh1_pm.C_scaffold_56000001	
pca2	TRIVE_53545	1.60e-01	0.30232118	jgi|Trive1|53545|e_gw1.56.8.1	
pca3	L50TH2P015R01416	1.49e-02	-0.7746587	L50TH2P015R01416	AJ911526
pca3	TRIVE_70952	5.85e-01	-0.09132282	jgi|Trive1|70952|fgenesh1_pg.C_scaffold_56000003	
pca4	TRIVE_53537	2.60e-01	0.19927485	jgi|Trive1|53537|e_gw1.56.24.1	
pca5	TRIVE_53528	2.86e-01	-0.16105261	jgi|Trive1|53528|e_gw1.56.11.1	
pca6	L52T3KP013R01168	2.32e-01	0.23519717	L52T3KP013R01168	AJ914873
pca6	L56TSTP015R01348	4.19e-01	-0.25470398	L56TSTP015R01348	AJ917970
pca6	TRIVE_53536	8.22e-01	0.04341014	jgi|Trive1|53536|e_gw1.56.29.1	
pca6	L56TSTP016R01528	8.64e-01	0.05801031	L56TSTP016R01528	AJ918137
pca6	L52T3KP015R01345	8.82e-01	0.03398337	L52T3KP015R01345	AJ915042
pca7	TRIVE_59982	3.35e-01	-0.32182377	jgi|Trive1|59982|fgenesh1_pm.C_scaffold_56000005	

Finally, since PCA1, PCA2, PCA4 and PCA7 have signal peptides suggesting secretion, we screened the results of two studies on secreted proteins upon plant interaction of *T. virens* [6,7], but none of the gene products of the *T.virens* PCA cluster was represented in the respective secretome.

These data support the assumption that the PCA cluster is indeed specifically induced upon early plant recognition and only plays a minor role at best during later stages of fungus-plant interaction. However, given the diverging cultivation conditions applied in the screened studies, it cannot be excluded that under *in vivo* conditions in soil the relevance of the PCA cluster extends to the colonization phase and may be specific to a fungal species and/or to a plant host.

Supplementary data 2

Indications that individual genes of the PCA cluster presumably originate from plants and bacteria

Our detailed investigation of genome sequences confirmed that the full PCA cluster is indeed only present in the Harzianum clade [8] of *Trichoderma*, but not in the more ancestral Trichoderma clade or in the evolutionarily younger Longibrachiatum clade. These findings suggest that the PCA cluster was gathered from other organisms by HGT, likely after the split of the Harzianum clade from the Longibrachiatum clade in *Trichoderma*.
We screened the identified genomic locations for the presence of the seven genes of the PCA cluster and found all of them in the same order and orientation in the Harzianum clade as well as in *Metarhizium* spp., which supports the hypothesis of HGT. In *P. fici* and *T. islandicus* the cluster is not complete, with *pca1* and *pca2* or *pca3* and *pca5* missing, respectively (Figure 5A). Individual genes of the cluster did show hits with blast searches in *Trichoderma* spp. outside of the Harzianum clade as well as in several other species, but they were not assembled in clusters in the respective genomes.

We therefore asked whether the *pca* genes might be present in the genomes of other *Trichoderma* clades, yet not assembled in clusters. We performed phylogenetic analysis using at least three blast hits (if present and e-value <e-10; otherwise all available) for the predicted protein sequences encoded by each *pca* gene in various organisms. This analysis allowed us to delineate, whether in these species the cluster simply lost several components during evolution or if those blast hits merely represent unrelated genes with similar function. We used representatives of the Harzianum species complex [9] (*T. harzianum, T. guizhouense, T. afroharzianum*) and the Harzianum clade (*T. pleuroticola, T. aggressivum* and *T. virens*) [9]. From the further clades we picked representatives from the Longibrachiatum clade (*T. reesei*), the Helicum clade (*T. helicum*) [10], the Brevicompactum clade (*T. brevicompactum*) [11] and the Trichoderma clade (*T. atroviride*) [12] as well as from *M. anisopliae, P. fici* and *T. islandicus* and other fungi having potential homologues in their genomes. Moreover, we added closely related sequences revealed as potential homologues from plants and bacteria as detected in blastp analysis using NCBI Blastp and excluding ascomycetes.

Interestingly, the latter blastp search on the NCBIdatabase yielded also highly related sequences to bacteria and plants for individual PCA proteins (Figure 5B), with *Quercus suber* (cork oak, plant) homologues as best hit for PCA1, PCA2 and PCA7, basidiomycete proteins for PCA3 and PCA5 as well as bacterial proteins for PCA4 and PCA6, which again suggested that the components of the cluster were assembled from different donor species.
This first exploratory phylogenetic analysis revealed the PCA proteins presumably connected by evolution and enabled us to distinguish those which clustered with proteins only similar, but not homologous to the PCA proteins and hence unrelated to the cluster. Indeed, the PCA homologues from species comprising the cluster formed a separate clade from those with similarity to the PCA sequences, but not organized in a cluster. In a second step we then used the closest hit of respective protein sequences from this sister clade for repeated phylogenetic analyses (Figure S1 A-G).

This analysis confirmed that only the *pca* genes from species having the cluster form a clade together, while *pca*-related genes scattered over the genome formed separate clades. Interestingly, *T. atroviride* seems to have homologues of PCA1 and PCA3 (Figure S1A, C), since the respective proteins cluster closely with the genes assembled in clusters in *T. harzianum* and not outside this clade. Additionally, we also found that hits from fungi outside ascomycetes, as well as those from plants or bacteria clustered among the fungal sequences or as sister clades hence confirming the blastp results. Consequently, acquisition of individual genes even from different kingdoms seems possible, although the highly conserved structure of the cluster in *Metarhizium*, *Trichoderma* and in part *P. fici* and *T. islandicus* suggests assembly of the cluster in one of these genera.

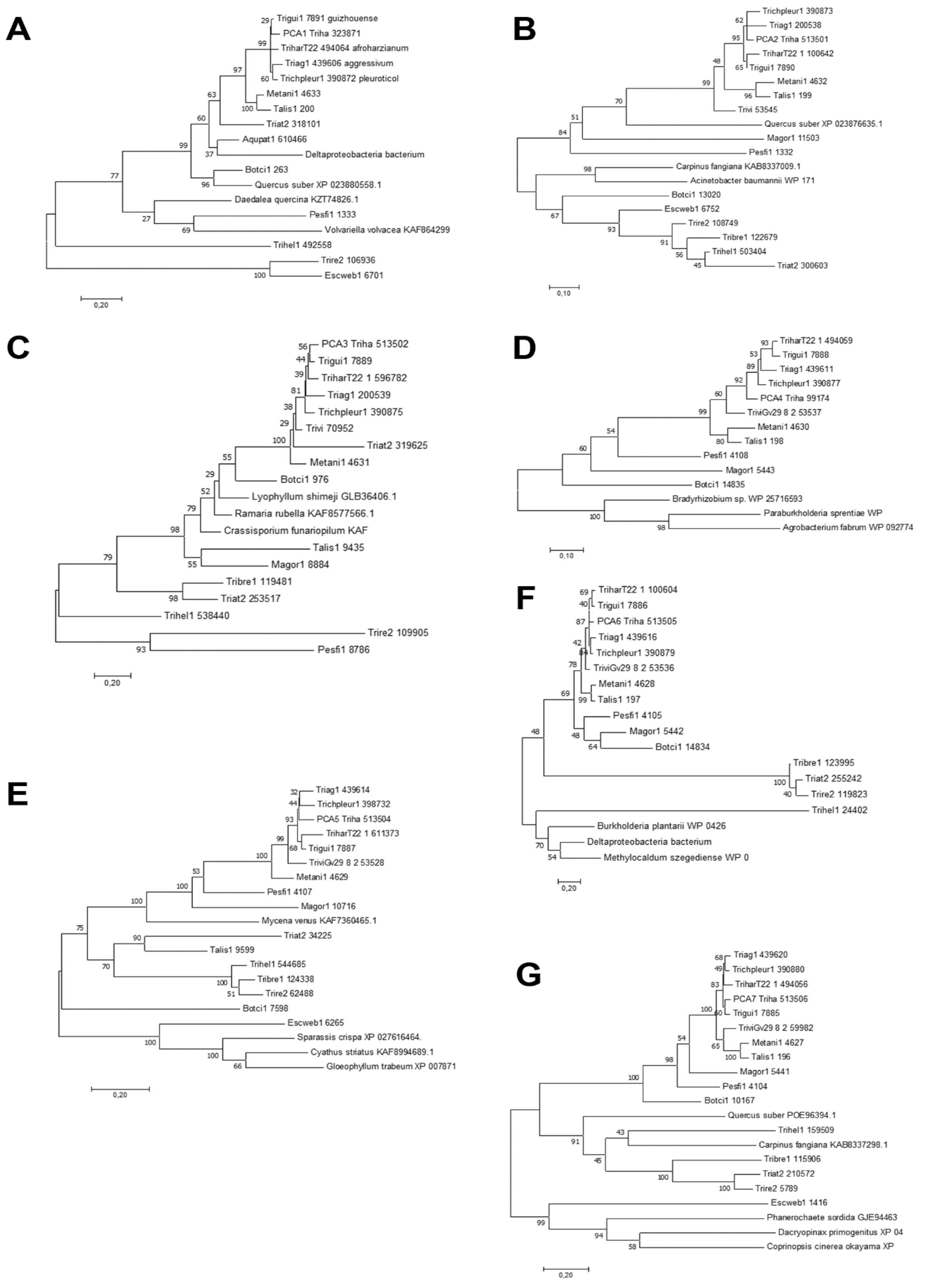

Figure S2. **Molecular phylogenetic analysis of the PCA cluster genes by the Maximum Likelihood method.** Maximum likelihood trees of homologues of (A) PCA1, (B) PCA2, (C) PCA3, (D) PCA4, (E) PCA5, (F) PCA6 and (G) PCA7 are shown. The evolutionary history was inferred by using the Maximum Likelihood method based on the JTT matrix-based model. The tree with the highest log likelihood is shown. The percentage of trees in which the associated taxa clustered together is shown next to the branches. Initial tree(s) for the heuristic search were obtained automatically by applying Neighbor-Join and BioNJ algorithms to a matrix of pairwise distances estimated using a JTT model, and then selecting the topology with superior log likelihood value. The trees are drawn to scale, with branch lengths measured in the number of substitutions per site. All positions with less than 95% site coverage were eliminated. That is, fewer than 5% alignment gaps, missing data, and ambiguous bases were allowed at any position.

The PCA cluster was not acquired by horizontal gene transfer

Phylogenetic analysis of predicted proteins within the PCA cluster as described above revealed some discordance with species phylogeny. Additionally, the unusual structure of the cluster, missing the typical biosynthetic polyketide synthases (PKS) or non-ribosomal peptide synthases (NRPS) demanded a closer look. Besides the PCA cluster, also a locus on a different scaffold comprising genes encoding an NRPS and a PKS could potentially contribute to biosynthesis of the metabolite associated with the PCA cluster. Consequently, we were interested whether this inconsistency is the result of horizontal gene transfer of part or all of the genes of the PCA cluster and whether the additional locus is concerned as well.

Using the package HGTphyloDetect [13], we investigated all protein sequences of the PCA cluster as well as the NRPS and PKS, which are regulated as well but located in a different genomic site. An alien index (AI) [13] of ≥ 45 was found to be a good indicator of foreign origin of a sequence [14]. For HGT among closely related organisms (e.g. eukaryote to eukaryote) the HGT index [13] was applied in order to capture potential HGT events for example from *Metarhizium* to *Trichoderma* as well.
We found no support for the hypothesis that either the cluster or the PKS- or NRPS-encoding gene in the second locus were acquired by HGT (supplementary table S1). Nevertheless, the finding of obviously closely related proteins in other fungi, plants or even bacteria (Figure 5C) was still puzzling. Therefore we performed the same analysis with the respective sequences from plants and bacteria. No HGT could be confirmed for sequences related to *pca3*, *pca5* and those from bacteria or other fungi. However, we found that *pca1*, *pca2* and *pca7* were indeed subject to horizontal gene transfer from fungi (*Botrytis* spp. and related species) to *Quercus suber* (supplementary table S1). In order to rule out that this result is merely caused by sequencing artifacts [15] in the published *Q. suber* sequence, we also tested the next best hits in plants for *pca7*. HGT was also detected for the *pca7* homologues in *Carpinus fangiana* and *Adiantum nelumboides* (supplementary table S1), hence rejecting the hypthesis of an artifact.

Interestingly, all individual genes of the PCA cluster have homologues in *Botrytis cinerea* (as evaluated using the JGI mycocosm genome databases for this species). However, the respective genes are not assembled in a cluster. Only *pca1* and *pca2* are next to each other in the genome. In *Q. suber* the homologues of *pca1*, *pca2* and *pca7* for which HGT is supported are not located in genomic vicinity, but on different scaffolds.

Supplementary table S5. Analysis of horizontal gene transfer using HGTphyloDetect.

	species	gene	related to	alien-index	e-value	HGT	predicted Donor
**PCA cluster**	*T. harzianum*	*pca1*		n.a.	-	no	-
	T. harzianum	*pca2*		n.a.	-	no	-
	T. harzianum	*pca3*		-55.91	-	no	-
	T. harzianum	*pca4*		34.67	-	no	-
	T. harzianum	*pca5*		n.a.	-	no	-
	T. harzianum	*pca6*		n.a.	-	no	-
	T. harzianum	*pca7*		n.a.	-	no	-
**PKS**	*T. harzianum*	XP_024768017		n.a.	-	no	-
**NRPS**	*T. harzianum*	XP_024768014		n.a.	-	no	-
**distant homolo-gues of PCA cluster genes**	***Quercus suber***	**XP_023880558.1**	***pca1***	**460.52**	**0.0**	**yes**	***Botrytis sp.***
	Carpinus fangiana	**KAB8337298.1**	***pca1***	**342.91**	**1.19e-149**	**yes**	***Coleo-phoma sp.***
	Adiantum nelumboides	**MCO5585497.1**	***pca1***	**460.52**	**0.0**	**yes**	***Meira spp.***
	Deltaproteo-bacteria bacterium	MAD86624.1	*pca1*	n.a.	-	no	-
	Daedalea quercina	KZT74826.1	*pca1*	-460.52	-	no	-
	Quercus suber	**XP_023876635.1**	***pca2***	**160.72**	**1.59e-70**	**yes**	***Botrytis sp.***
	Ramaria rubella	KAF8577566.1	*pca3*	0.00	-	no	-
	Mycena venus	KAF7360465.1	*pca5*	-460.52	-	no	-
	Quercus suber	**POE96394.1**	***pca7***	**460.52**	**0.0**	**yes**	***Zasmidium sp.***

n.a. not available i.e. calculation of alien index not successful, no support for HGT

The PCA cluster is largely subject to balancing evolution

The results of our phylogenetic analysis together with HGT analysis suggested that PCA cluster genes may have been lost during evolution in many fungal species, including *Trichoderma* spp. outside the Harzianum cluster. Therefore we were interested, whether evolutionary pressure may have caused this effect. To distinguish between random (neutral) evolution and non-random evolution like directional selection or balancing selection, we applied the Tajima's D test [16]. In case of randomly involving sequences, no effect on the fitness or survival of an organism is expected and the frequenies of such neutral mutations would fluctuate through genetic drift. Mutations affecting the fitness or survival of an organism – such as for example lethal mutations – are non-neutral and under selection.
Calculation of Tajimas D from individual conding regions did not yield statistically significant results for synonymous sites, non-synonymous sites or overall except for synonymous sites of *pca5*. The Tajimas D (non-syn/syn) ratio shows tendencies towards balancing selection or a balance between mutation and genetic drift, for *pca6* and *pca7*, the negative values support a hypothesis of positive selection (supplementary table S2).
Hence we consider it likely, that the PCA cluster has a neutral or positive effect in species where it was retained, potentially in combination with the respective ecological niche, like for *T. harzianum*. Such a positive effect would also be in agreement with the acquisition of *pca7* by plants from fungi, although the benefit of this gene for plants remains to be determined.

Table S6. Tajimas D for the genes of the PCA cluster

gene	Tajimas D (nonsyn/syn) ratio	selection
pca1	-0,5636	balance between mutation and genetic drift
pca2	21,22982	balancing selection
pca3	25,01958	balancing selection
pca4	0,22122	balance between mutation and genetic drift
pca5	**0,03811**	**balance between mutation and genetic drift**
pca6	-4,45727	positive selection
pca7	-13,21566	positive selection

Supplementary table S7 – Blastp analysis of PCA cluster genes in selected genomes (as shown in Figure 5A)

	PCA1		PCA2		PCA3		PCA4		PCA5		PCA6		PCA7	
**strain**	**% ID**	**e-value**	**% ID**	**e-value**	**% ID**	**e-value**	**% ID**	**e-value**	**% ID**	**e-value**	**% ID**	**e-value**	**% ID**	**e-value**
Trichoderma afroharzianum T22	88.5	0.0	91.0	9.11E-82	80.6	0.0	88.6	0.0	82.5	1.06E-176	90.4	5.68E-140	91.0	0.0
Trichoderma guizhouense NJAU4742	90.9	0.0	95.3	2.27E-81	87.0	0.0	90.9	0.0	88.0	0.0	92.5	1.11E-143	93.8	0.0
Trichoderma aggressivum SZM3109	89.0	0.0	89.6	8.98E-82	80.3	0.0	91.1	0.0	86.1	0.0	87.9	3.67E-136	92.8	0.0
Trichoderma pleuroticola CBS121217	88.7	0.0	91.1	9.10E-82	82.9	0.0	92.0	0.0	86.4	0.0	91.2	2.46E-141	93.4	0.0
Trichoderma viren s Gv29-8	82.6	0.0	83.7	4.13E-77	77.8	0.0	87.0	0.0	83.8	0.0	88.7	1.72E-138	85.2	0.0
Metarhizium anisopliae ARSEF549	77.0	0.0	67.0	8.04E-48	74.3	0.0	80.6	0.0	81.2	0.0	81.2	6.83E-125	81.4	0.0
Pestalotiopsis fici W106-1	-	-	-	-	-	-	61.0	2.05E-64	60.8	2.03E-134	69.7	4.01E-108	67.8	0.0
Talaromyces islandicus WF3812	78.7	0.0	66.3	3.25E-71	-	-	82.3	0.0	-	-	80.08	1.59E-126	81.2	0.0

Supplementary table S8. Oligonucleotides used in this study

**Name**	**Function**	**Sequence 5' - 3'**	**Protein ID**	**targtet gene**
Bpca1del5F	creation of 5' flank of deletion cassette	5’GTAACGCCAGGGTTTTCCCAGTCACGACGATGGTGGTGATTGTTGTG 3‘	323871	*pca1*
Bpca5del5R	creation of 5' flank of deletion cassette	5’ATCCACTTAACGTTACTGAAATCTCCAACGGTAAATGCGTTTCAAAG 3‘	323871	*pca1*
Bpca1del3F	creation of 3' flank of deletion cassette	5’CTCCTTCAATATCATCTTCTGTCTCCGACATTAAATGATACACAGGCTG 3‘	323871	*pca1*
Bpca1del3R	creation of 3' flank of deletion cassette	5’GCGGATAACAATTTCACACAGGAAACAGCCATTGTCATCTGCAGTAGAC 3‘	323871	*pca1*
Bpca1screF	confirmation of deletion	5’GGATGGACCTTACCCTTTATCG 3‘	323871	*pca1*
Bpca1screR	confirmation of deletion	5’ACCACAAACGAGTGCTGAAATC 3‘	323871	*pca1*
Bpca5del5F	creation of 5' flank of deletion cassette	5’GTAACGCCAGGGTTTTCCCAGTCACGACGTTGTCCGTTGTCCTATGGC 3‘	513504	*pca5*
Bpca5del5R	creation of 5' flank of deletion cassette	5’ATCCACTTAACGTTACTGAAATCTCCAACATCGCTTATTCGTTCGCAG 3‘	513504	*pca5*
Bpca5del3F	creation of 3' flank of deletion cassette	5’CTCCTTCAATATCATCTTCTGTCTCCGACGGTCCATTTGATAATAGAGAAG 3‘	513504	*pca5*
Bpca5del3R	creation of 3' flank of deletion cassette	5’GCGGATAACAATTTCACACAGGAAACAGCCTATTCACACCCAGAGCAC 3‘	513504	*pca5*
Bpca5screF	confirmation of deletion	5’CCGACGCAGGAAAGAAAC 3‘	513504	*pca5*
Bpca5screR	confirmation of deletion	5’ACAGATGTAGACGCAGCTGG 3‘	513504	*pca5*

References

1. Barrett, T. *et al.* (2013) NCBI GEO: archive for functional genomics data sets--update. *Nucleic Acids Res* 41, D991-995. 10.1093/nar/gks1193
2. Moran-Diez, M.E. *et al.* (2015) Host-specific transcriptomic pattern of *Trichoderma virens* during interaction with maize or tomato roots. *BMC Genomics* 16, 8. 10.1186/s12864-014-1208-3
3. Malinich, E.A. *et al.* (2019) Differential expression analysis of *Trichoderma virens* RNA reveals a dynamic transcriptome during colonization of *Zea mays* roots. *BMC Genomics* 20, 280. 10.1186/s12864-019-5651-z
4. Vizcaino, J.A. *et al.* (2007) Generation, annotation, and analysis of ESTs from four different *Trichoderma* strains grown under conditions related to biocontrol. *Appl Microbiol Biotechnol* 75, 853-862
5. Rubio, M.B. *et al.* (2012) Comparative study of *Trichoderma* gene expression in interactions with tomato plants using high-density oligonucleotide microarrays. *Microbiology* 158, 119-128. 10.1099/mic.0.052118-0
6. Lamdan, N.L. *et al.* (2015) Secretome of *Trichoderma* interacting with maize roots: role in induced systemic resistance. *Mol Cell Proteomics* 14, 1054-1063. 10.1074/mcp.M114.046607
7. Nogueira-Lopez, G. *et al.* (2018) The apoplastic secretome of *Trichoderma virens* during interaction with maize roots shows an inhibition of plant defence and scavenging oxidative stress secreted proteins. *Front Plant Sci* 9, 409. 10.3389/fpls.2018.00409
8. Atanasova, L. *et al.* (2013) Two hundred *Trichoderma* species recognized on the basis of molecular phylogeny. In *Trichoderma - Biology and Applications* (Mukherjee, P.K. *et al.*, eds), pp. 10-42, CAB International
9. Chaverri, P. *et al.* (2015) Systematics of the *Trichoderma harzianum* species complex and the re-identification of commercial biocontrol strains. *Mycologia* 107, 558-590. 10.3852/14-147
10. Jaklitsch, W.M. and Voglmayr, H. (2015) Biodiversity of *Trichoderma* (Hypocreaceae) in Southern Europe and Macaronesia. *Stud Mycol* 80, 1-87. 10.1016/j.simyco.2014.11.001
11. Degenkolb, T. *et al.* (2008) The *Trichoderma brevicompactum* clade: a separate lineage with new species, new peptaibiotics, and mycotoxins. *Mycological Progress* 7, 177-219. 10.1007/s11557-008-0563-3
12. Kubicek, C.P. *et al.* (2019) Evolution and comparative genomics of the most common *Trichoderma* species. *BMC Genomics* 20, 485. 10.1186/s12864-019-5680-7
13. Yuan, L. *et al.* (2023) HGTphyloDetect: facilitating the identification and phylogenetic analysis of horizontal gene transfer. *Brief Bioinform* 24. 10.1093/bib/bbad035
14. Gladyshev, E.A. *et al.* (2008) Massive horizontal gene transfer in bdelloid rotifers. *Science* 320, 1210-1213. 10.1126/science.1156407
15. Martin, W.F. (2017) Too much eukaryote LGT. *Bioessays* 39. 10.1002/bies.201700115
16. Tajima, F. (1989) Statistical method for testing the neutral mutation hypothesis by DNA polymorphism. *Genetics* 123, 585-595. 10.1093/genetics/123.3.585
